# Protosappanin A Protects DOX‐Induced Myocardial Injury and Cardiac Dysfunction by Targeting ACSL4/FTH1 Axis‐Dependent Ferroptosis

**DOI:** 10.1002/advs.202310227

**Published:** 2024-07-10

**Authors:** Jingxuan Cui, Yujia Chen, Qiannan Yang, Peng Zhao, Mian Yang, Xiaoqi Wang, Ge Mang, Xiangyu Yan, Di Wang, Zhonghua Tong, Penghe Wang, Yingjin Kong, Naixin Wang, Dongni Wang, Nana Dong, Mingyang Liu, Mingyan E, Maomao Zhang, Bo Yu

**Affiliations:** ^1^ Department of Cardiology The Second Affiliated Hospital of Harbin Medical University Harbin 150086 China; ^2^ The Key Laboratory of Myocardial Ischemia Harbin 150086 China; ^3^ State Key Laboratory of Frigid Zone Cardiovascular Disease Harbin 150086 China; ^4^ Department of Cardiology Daping Hospital The Third Military Medical Chongqing 400000 China; ^5^ Department of Cardiology The Fourth Affiliated Hospital of Harbin Medical University Harbin 150086 China; ^6^ Department of Thoracic Radiotherapy Harbin Medical University Cancer Hospital Harbin China

**Keywords:** doxorubicin‐induced cardiomyopathy, ferroptosis, molecular targeted therapy, myocardial injury, protosappanin A

## Abstract

Doxorubicin (DOX) is an effective anticancer agent, but its clinical utility is constrained by dose‐dependent cardiotoxicity, partly due to cardiomyocyte ferroptosis. However, the progress of developing cardioprotective medications to counteract ferroptosis has encountered obstacles. Protosappanin A (PrA), an anti‐inflammatory compound derived from hematoxylin, shows potential against DOX‐induced cardiomyopathy (DIC). Here, it is reported that PrA alleviates myocardial damage and dysfunction by reducing DOX‐induced ferroptosis and maintaining mitochondrial homeostasis. Subsequently, the molecular target of PrA through proteome microarray, molecular docking, and dynamics simulation is identified. Mechanistically, PrA physically binds with ferroptosis‐related proteins acyl‐CoA synthetase long‐chain family member 4 (ACSL4) and ferritin heavy chain 1 (FTH1), ultimately inhibiting ACSL4 phosphorylation and subsequent phospholipid peroxidation, while also preventing FTH1 autophagic degradation and subsequent release of ferrous ions (Fe^2+^) release. Given the critical role of ferroptosis in the pathogenesis of ischemia‐reperfusion (IR) injury, this further investigation posits that PrA can confer a protective effect against IR‐induced cardiac damage by inhibiting ferroptosis. Overall, a novel pharmacological inhibitor is unveiled that targets ferroptosis and uncover a dual‐regulated mechanism for cardiomyocyte ferroptosis in DIC, highlighting additional therapeutic options for chemodrug‐induced cardiotoxicity and ferroptosis‐triggered disorders.

## Graphical abstract

The diagram depicts the protective mechanism of protosappanin A (PrA) on doxorubicin (DOX)‐induced cardiomyopathy. Briefly, PrA binds directly to acyl‐CoA synthetase long‐chain family member 4 (ACSL4) and ferritin heavy chain 1 (FTH1), blocking the enzymatic activation and phosphorylation of ACSL4 which catalyzes metabolism of polyunsaturated fatty acids (PUFA) and interfering with the nuclear receptor coactivator 4 (NCOA4)‐FTH1 interaction and ferritinophagy process. 

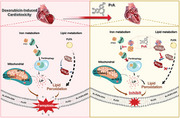

## 1 Introduction

Anthracyclines such as doxorubicin (DOX) are highly effective in treating solid tumors and hematologic malignancies.^[^ ^1^ ^]^ Nonetheless, the cardiotoxic effects of chemotherapeutic drugs severely affect a patient's quality of life, manifesting as arrhythmia, irreversible ventricular dysfunction, and heart failure after long‐term exposure to the medication.^[^ ^2^ ^]^ The occurrence of DOX‐induced cardiomyopathy (DIC) commonly signifies a poor prognosis. The incidence of DOX‐related heart failure was reported to be nearly 25% at a cumulative dose of 700 mg m^−2^.^[^ ^3^ ^]^ Although the underlying pathogenesis of DIC has been elucidated, specific prevention and treatment strategies are still lacking.

Ferroptosis is a novel cell death process that results from the peroxidation of membrane phospholipids.^[^ ^4^ ^]^ Unlike other forms of cell death, ferroptosis principally manifests as mitochondrial disorders, abnormal iron metabolism, excessive lipid peroxidation, and glutathione peroxidase 4 (GPX4)/glutathione (GSH) axis imbalance.^[^ ^5^ ^]^ Emerging evidence indicates that ferroptosis is closely associated with DOX‐mediated cardiac dysfunction.^[^ ^6^ ^]^ Recently, ferroptotic phenotypes were identified in DOX‐challenged cardiomyocytes.^[^ ^7^ ^]^ Based on the above findings, the development of targeted medications for ferroptosis has the potential to mitigate myocardial damage caused by DOX and improve clinical outcomes for patients.

Dexrazoxane (DXZ) is a widely approved cardioprotective agent for the treatment of anthracycline‐mediated cardiotoxicity that targets chelated intracellular ferrous ions and prevents Fenton reaction‐mediated ferroptosis.^[^ ^8^ ^]^ However, the therapeutic effect of DXZ on cancer survival is controversial because of side effects such as myelosuppression and resistance to chemodrug‐mediated antitumor effects.^[^ ^9^ ^]^ Therefore, it is important to develop safe and effective therapeutic strategies to combat chemotherapy drug‐induced cardiotoxicity. Protosappanin A (PrA), an active constituent extracted from traditional Chinese hematoxylin, has been associated with multiple pharmacological properties, including antioxidative, anti‐inflammatory, and anti‐apoptotic activities.^[^ ^10^ ^]^ Our previous research demonstrated that PrA could exert an immunosuppressive effect on cardiac transplantation and autoimmune myocarditis via NF‐κB and Akt/mTOR.^[^ ^11^ ^]^ Despite recent advances, the role of PrA in DIC and its relationship with ferroptosis remain unclear.

Therefore, our study aimed to investigate the impact of PrA on DIC and its mechanism, with a focus on ferroptosis. We found that PrA protected against DOX‐induced myocardial injury and alleviated ferroptosis‐induced mitochondrial dysfunction. Mechanistically, PrA binds directly to acyl‐CoA synthetase long‐chain family member 4 (ACSL4) and ferritin heavy chain 1 (FTH1), preventing ACSL4 phosphorylation and FTH1 autophagic degradation, thereby inhibiting cardiomyocyte ferroptosis. Since cardiac dysfunction induced by ischemia‐reperfusion (I/R) can be alleviated by inhibiting ferroptosis,^[^ ^12^ ^]^ we further confirmed that PrA mitigated I/R‐mediated ferroptosis and cardiac injury. Collectively, our data shed new light on pharmacological approaches targeting ferroptosis for the clinical treatment of chemodrug‐induced cardiotoxicity and other cardiovascular‐related conditions.

## 2 Results

### 2.1 PrA Alleviates DOX‐Induced Myocardial Injury and Cardiac Dysfunction

**Figure 1:**
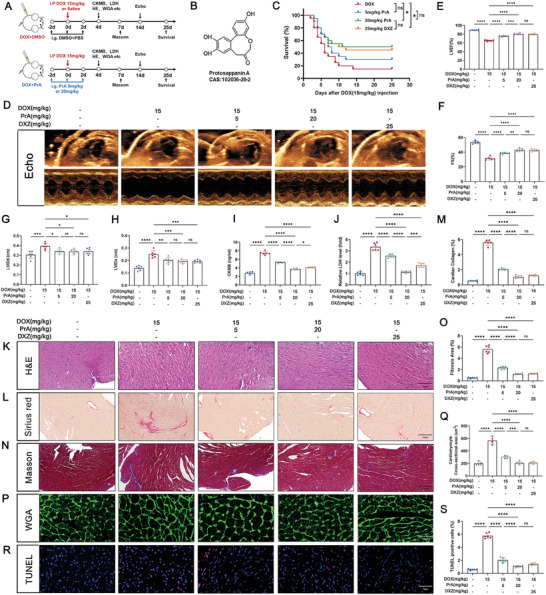
PrA alleviates DOX‐induced cardiac injury. A) Schematic diagram of animal experiment process. Briefly, mice were treated with PrA (5 or 20 mg kg^−1^, i.g.), DXZ (an iron chelating agent, 25 mg kg^−1^, i.p.) or DMSO with PBS on day 0, day 2, and day 4, followed by DOX (20 mg kg^−1^, i.p.) on day 2, while mice were injected with saline only as a control treatment on day 2. B) The chemical structure of the PrA. C) Kaplan‐Meier survival curves of mice in each group (*n* = 20 per group). D) Representative echocardiographic images showing the cardiac function of mice in each group. E–H) Quantitative analysis of LVEF, FS, LVIDd, and LVIDs (*n* = 6 per group). I,J) Serum CK‐MB (fivefold dilution) and LDH levels were measured in each group (*n* = 6 per group). K) Representative images of H&E staining (*n* = 6 per group, scale bar = 100 µm). L,M) Representative images and quantitative analysis of Sirius red staining (*n* = 6 per group, scale bar = 100 µm). N,O) Representative images and quantitative analysis of Masson's trichrome staining (*n* = 6 per group, scale bar = 100 µm). P) Representative images and Q) quantitative analysis of WGA staining (*n* = 6 per group, scale bar = 20 µm). R) Representative images and S) quantitative analysis of TUNEL staining (*n* = 6 per group, scale bar = 20 µm). The data presented in panel J was normalized. Summary data are presented as the mean ± SEM. Statistical significance was calculated using C) the log‐rank (Mantel‐Cox) test and E–J,K,M,O, Q,S) one‐way ANOVA with Tukey's multiple comparisons test. *p < 0.05, **p < 0.01, ***p < 0.001, ****p < 0.0001. Abbreviations: CK‐MB, creatine kinase‐MB; DMSO, dimethylsulfoxide; DOX, doxorubicin; DXZ, dexrazoxane; FS, left ventricular fractional shortening; i.g., intragastric; i.p., intraperitoneal; LDH, lactate dehydrogenase; LVEF, left ventricular ejection fraction; LVIDd, left ventricular internal dimension in diastole; LVIDs, left ventricular internal dimension in systole; PBS, phosphate buffer saline; PrA, protosappanin A.

To investigate the role of PrA in DOX‐induced cardiotoxicity, a DIC murine model was established as described and treated with PrA (5 mg kg^−1^, 20 mg kg^−1^) or DXZ for the indicated days (**Figure** **1**A,B). We estimated the survival rates of these mice. Similar to DXZ, PrA significantly improved the survival rate of DOX‐treated mice in a dose‐dependent manner (Figure 1C). Echocardiographic assessment showed a significant decline in cardiac function in DIC mice compared to the control group, primarily reflected in the left ventricular ejection fraction (LVEF), left ventricular fractional shortening (FS), left ventricular internal dimension in diastole (LVIDd), and left ventricular internal dimension in systole (LVIDs) parameters. In contrast, partial recovery of cardiac function was observed following treatment with DXZ or increasing doses of PrA, indicating a protective effect against DOX‐induced cardiac dysfunction (Figure 1D–H). We evaluated the effect of PrA on DOX‐induced myocardial damage by analyzing myocardial injury biomarkers and cardiac histopathological changes. Biochemical analysis revealed that PrA treatment significantly inhibited the DOX‐induced elevation of serum creatine kinase‐MB (CK‐MB) and lactate dehydrogenase (LDH) levels in a dose‐response manner (Figure 1I,J). Hematoxylin and eosin (H&E) staining showed severe inflammatory infiltrates in DIC mice, which were alleviated by increasing the concentration of PrA (Figure 1K). Moreover, PrA dose‐dependently inhibited DOX‐induced cardiomyocyte hypertrophy and myocardial fibrosis in mice, as determined by wheat germ agglutinin (WGA), Sirius Red, and Masson's trichrome staining (Figure 1L–Q). TUNEL staining confirmed that DOX‐induced myocardial DNA damage was ameliorated by PrA treatment (Figure 1R,S). Notably, a high PrA concentration of 20 mg kg^−1^ exhibited cardioprotective efficacy in DIC, partially exceeding that of DXZ, mainly reflecting a better suppression of DOX‐mediated serum CK‐MB and LDH levels (Figure 1I,J). Considering that DOX is commonly used in the treatment of breast cancer, we retested the above indicators utilizing female mice and obtained results similar to those of male mice (Figure S1A–H,N–P, Supporting Information). Taken together, our data suggest that PrA attenuates DOX‐induced cardiac injury and prevents cardiac dysfunction.

### 2.2 PrA Inhibits DOX‐Induced Cardiac Ferroptosis

**Figure 2:**
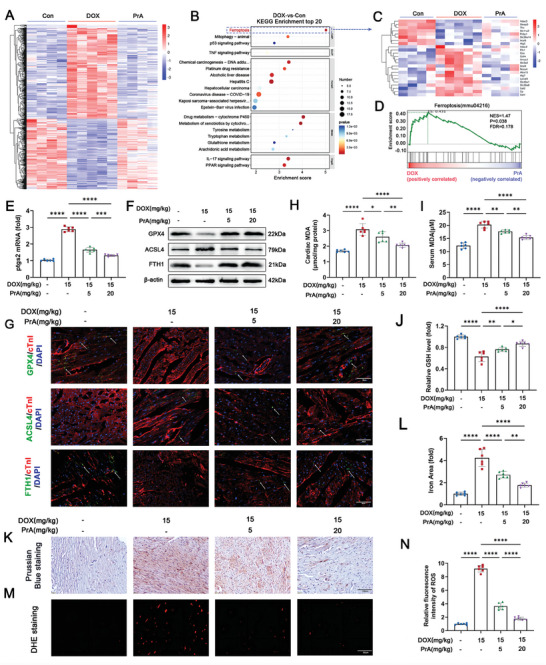
PrA ameliorates DOX‐induced ferroptosis in heart tissue. A–D) RNA‐seq analysis reveals the DEGs in murine hearts from three groups: control (*n* = 4 per group), DOX (15 mg kg^−1^, i.p.; *n* = 5 per group), and DOX + PrA (20 mg kg^−1^, i.g.; *n* = 4 per group), as mentioned in Figure 1A. A) Heat map representing the significantly regulated genes detected in RNA‐seq analysis of murine heart tissue. Gene expressions were normalized with row Z‐score. B) KEGG pathway enrichment analysis in DOX‐treated murine heart compared with control murine heart. C) Heat map representing the ferroptosis‐related genes detected in RNA‐seq analysis of murine heart tissue. Gene expressions were normalized with row Z‐score. D) GSEA of regulated genes in DOX with PrA‐treated murine heart compared with DOX‐treated murine heart. E–N) Mice were randomly divided into four groups (*n* = 6 per group): control, DOX, DOX+PrA (5 mg kg^−1^), and DOX+PrA (20 mg kg^−1^). E) Relative mRNA levels of Ptgs2 in murine hearts. F) Cardiac protein expression of GPX4, ACSL4, and FTH1 were measured by immunoblotting. G) Representative images of cofluorescent immunohistochemistry staining for cTnT with GPX4 (green), ACSL4 (green), and FTH1 (green) in mice; the white arrows indicated positive areas. H) Cardiac MDA, I) serum MDA, and J) cardiac GSH levels were measured. K) Representative images and L) % area of Prussian blue staining with DAB enhancement (scale bar:100 µm). M,N) Representative images and quantifying fluorescent immunohistochemistry staining for DHE (scale bar: 20 µm). E,J,L,N) Some of the data was normalized. Summary data are presented as the mean ± SEM. Statistical significance was calculated using one‐way ANOVA with Tukey's multiple comparisons test. *p < 0.05, **p < 0.01, ***p < 0.001, ****p < 0.0001. Abbreviations: DOX, doxorubicin; i.p., intraperitoneal; i.g., intragastric; PrA, protosappanin A.

To elicit the molecular mechanism underlying the protective effects of PrA on DIC, RNA‐sequencing was conducted on murine hearts from three groups: control, DOX, and DOX combined with PrA treatment (20 mg kg^−1^). The differentially expressed genes (DEGs) for the indicated comparison groups are presented in **Figure** **2**A and Figure S2A,B (Supporting Information). As revealed by the Kyoto Encyclopedia of Genes and Genomes (KEGG) analysis (Figure 2B), the ferroptosis pathway was the top‐ranked enrichment among these DEGs in the DOX‐conditioned group compared to the control group, supporting the close relationship between ferroptosis and DIC. Conversely, PrA treatment remarkably reduced ferroptosis‐related DEGs enrichment induced by DOX (Figure 2C and Figure S2C, Supporting Information). Additionally, gene set enrichment analysis (GSEA) confirmed that ferroptosis was upregulated by DOX but downregulated by PrA (Figure 2D), implicating that PrA may protect against DIC by inhibiting ferroptosis.

To validate the data of RNA‐sequencing, we assessed the impact of PrA on ferroptosis in DIC mice. The expression of the ferroptosis marker Ptsg2 was analyzed in mouse hearts using polymerase chain reaction (PCR). As expected, PrA dose‐dependently reversed the Ptsg2 gene expression induced by DOX (Figure 2E and Figure S1I, Supporting Information). Subsequently, we evaluated the cardiac abundance of essential regulatory proteins linked to ferroptosis in mice, including GPX4, ACSL4, and FTH1. Our results showed that ACSL4 was more abundant, whereas FTH1 and GPX4 were less abundant in the hearts of DIC mice than in those of control mice. PrA administration reversed the DOX‐mediated alteration in these protein levels in the mice (Figure 2F and Figures S2D–F and S1J–M, Supporting Information). Immunofluorescence further supported the immunoblotting findings (Figure 2G and Figure S2G–I, Supporting Information).

Given that iron overload, lipid peroxidation, and intracellular reactive oxygen species (ROS) accumulation are critical features of ferroptosis, we further examined the levels of the lipid peroxidation products malondialdehyde (MDA) and glutathione (GSH) in the aforementioned conditioned mice. Compared to the control group, decreased GSH cardiac levels and elevated MDA cardiac and serum levels were observed in DIC mice. In contrast, PrA reversed the DOX‐induced changes in MDA and GSH levels (Figure 2H–J). Moreover, total iron and ROS levels were significantly increased in heart tissues after DOX administration, and PrA treatment abrogated these DOX‐induced results (Figure 2K–N). Taken together, our data imply that PrA negatively regulates cardiac ferroptosis in DIC mice.

### 2.3 PrA Attenuates DOX‐Induced Cardiomyocyte Ferroptosis and Maintains Mitochondrial Function

**Figure 3:**
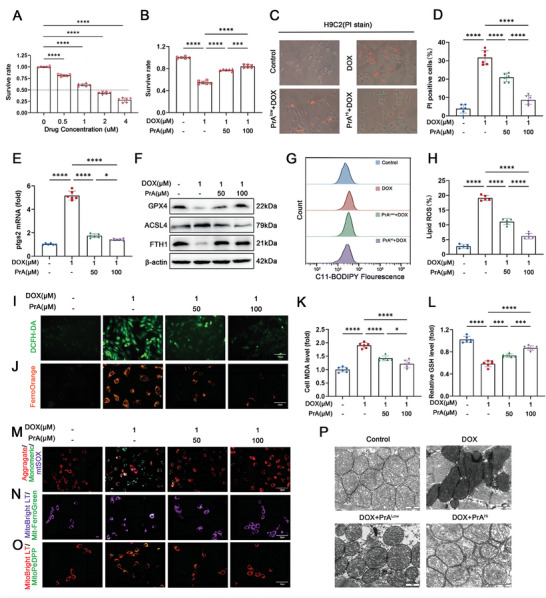
PrA protects H9c2 cells against DOX‐triggered iron accumulation, ROS production, lipid peroxidation and alleviates mitochondrial dysfunction. A) The effect in different concentrations of DOX‐induced cell death for 24 h, cell viability was detected by CCK8 assay (*n*  = 6 per group). B) The protective effect of PrA treatment on DOX‐induced cell death in cultured H9c2 cells under control conditions, in the presence of DOX (1 × 10^−6^ m, 24 h) or PrA (50 × 10^−6^ m or 100 × 10^−6^ m, 30 h), cell viability was detected by CCK8 assay (*n*  = 6 per group). H9c2 cells were pretreated with PrA in different concentrations for 6 h and then stimulated with 1 × 10^−6^ m DOX for 24 h. DMSO was used as vehicle control. Whole cells were used for the following assay. C) Representative images of PI staining and D) the percentage of PI‐positive cells (black and white: phase contract; red: PI staining, scale bar  =  100 µm, *n* = 6 per group). E) Relative mRNA levels of Ptgs2 (*n* = 6 per group). F) Protein expression of GPX4, ACSL4, and FTH1 was measured by immunoblotting (*n* = 6 per group). G,H) Lipid ROS generation and quantitative analysis were captured by C11‐BODIPY staining coupled with flow cytometry (*n* = 5 per group). I) Representative DCFH‐DA staining image (green, scale bar = 100 µm). J) Representative fluorescent images of cytoplasmic Fe^2+^ stained with FerroOrange (orange, scale bar = 20 µm). Quantitative analysis of K) MDA level and L) GSH level (*n* = 6 per group). M) JC‐1 staining assessed membrane potential and mtSOX Deep Red staining detected mitochondrial superoxide level (red, aggregates; green, monomersscale; purple, mtSOX Deep Red; scale bar = 20 µm). N) Representative fluorescent images of mitochondrial iron using Mito‐FerroGreen (MFG) in H9c2 cells. MitoBright LT Deep Red stains the mitochondria (green, MFG; purple, MitoBright LT Deep Red; scale bars: 20 µm). O) Representative fluorescent images of mitochondrial LPs using MitoPeDPP in H9c2 cells. MitoBright LT Deep Red stains the mitochondria (green, MitoPeDPP; red, MitoBright LT Deep Red; scale bar = 20 µm). P) Electron microscopy of mitochondria in mice hearts (scale bar =  2 µm). A,B,E,K,L) Some of the data was normalized. Summary data are presented as the mean ± SEM. Statistical significance was determined using A) multiple unpaired 2‐tailed Student *t*‐tests and B,D,E,H,K–L) one‐way ANOVA with Tukey's multiple comparisons tests. *p < 0.05, **p < 0.01, ***p < 0.001, ****p < 0.0001. Abbreviations: DMSO, dimethylsulfoxide; DOX, doxorubicin; PrA, protosappanin A; ROS, reactive oxygen species.

We explored the cytoprotective mechanism of PrA against ferroptosis in rat H9c2 cells in vitro. H9C2 cells were cultured with different concentrations of DOX (0 × 10^−6^, 0.5 × 10^−6^, 1 × 10^−6^, 2 × 10^−6^, and 4 × 10^−6^ m) for 24 h, resulting in a significant reduction in cell viability (**Figure** **3**A). PrA treatment showed almost the same impact on cardiomyocyte viability with no cytotoxicity at 50 × 10^−6^ m and 100 × 10^−6^ m in the absence of DOX exposure (Figure S3A, Supporting Information). Based on the above cell viability results, we selected concentrations of 1 × 10^−6^ m DOX and 50 × 10^−6^ m, and 100 × 10^−6^ m PrA for subsequent experiments. According to the results of Cell Counting Kit 8 (CCK8) and propidium iodide (PI) fluorescence staining assays, PrA was shown to dose‐dependently reduce DOX‐induced cardiomyocyte damage in vitro, demonstrating cytoprotective effects (Figure 3B–D). Consistent with the in vivo results, PrA treatment reversed the levels of ferroptosis‐related parameters in DOX‐conditioned cardiomyocytes, including the Ptgs2 gene (Figure 3E), GPX4, ACSL4, and FTH1 proteins (Figure 3F and Figure S3B–D, Supporting Information), lipid and intracellular ROS levels (Figure 3G–I and Figure S3E, Supporting Information), intracellular ferrous ions (Fe^2+^) content (Figure 3J and Figure S3F, Supporting Information), and lipid peroxidative products MDA and GSH (Figure 3K,L). These results indicate that PrA effectively reduced the susceptibility of DOX‐treated cardiomyocytes to ferroptosis in vitro.

Mitochondria‐dependent iron accumulation and lipid peroxidation are crucial factors in DOX‐induced ferroptosis.^[^ ^13^ ^]^ As previously reported, we found that Fe^2+^ and lipid peroxide content increased mainly in cultured cardiomyocyte mitochondria during DOX treatment. However, PrA treatment abolished this phenomenon induced by DOX (Figure 3N,O and Figure S3H,I,J–M, Supporting Information). We further monitored the critical indices reflecting mitochondrial function,^[^ ^14^ ^]^ including mitochondrial membrane potential (ΔΨ*m*) and mitochondrial‐derived ROS (mitoROS) production, under all the aforementioned conditions. As depicted in Figure 3M and Figure S3G,J,K (Supporting Information), DOX‐treated cardiomyocytes exhibited decreased ΔΨm and increased mitochondrial superoxide accumulation compared to the control group. Conversely, PrA intervention reversed these changes induced by DOX. Similarly, the protective effects of PrA on DOX‐induced ferroptosis indices and mitochondrial damage were also observed in primary murine neonatal cardiomyocytes (Figure S4, Supporting Information). Additionally, the structural and morphological characteristics of mitochondria were observed using electron transmission microscopy. As shown in Figure 3P, it was revealed that DOX exposure resulted in a shrinkage of mitochondria and increased mitochondrial membrane density, whereas PrA treatment improved DOX‐mediated abnormal features in a dose‐dependent manner in murine heart tissue. These results demonstrate the protective effect of PrA against DOX‐induced mitochondria‐dependent ferroptosis.

### 2.4 ACSL4 and FTH1 Are Identified as Direct PrA Binding Targets

**Figure 4:**
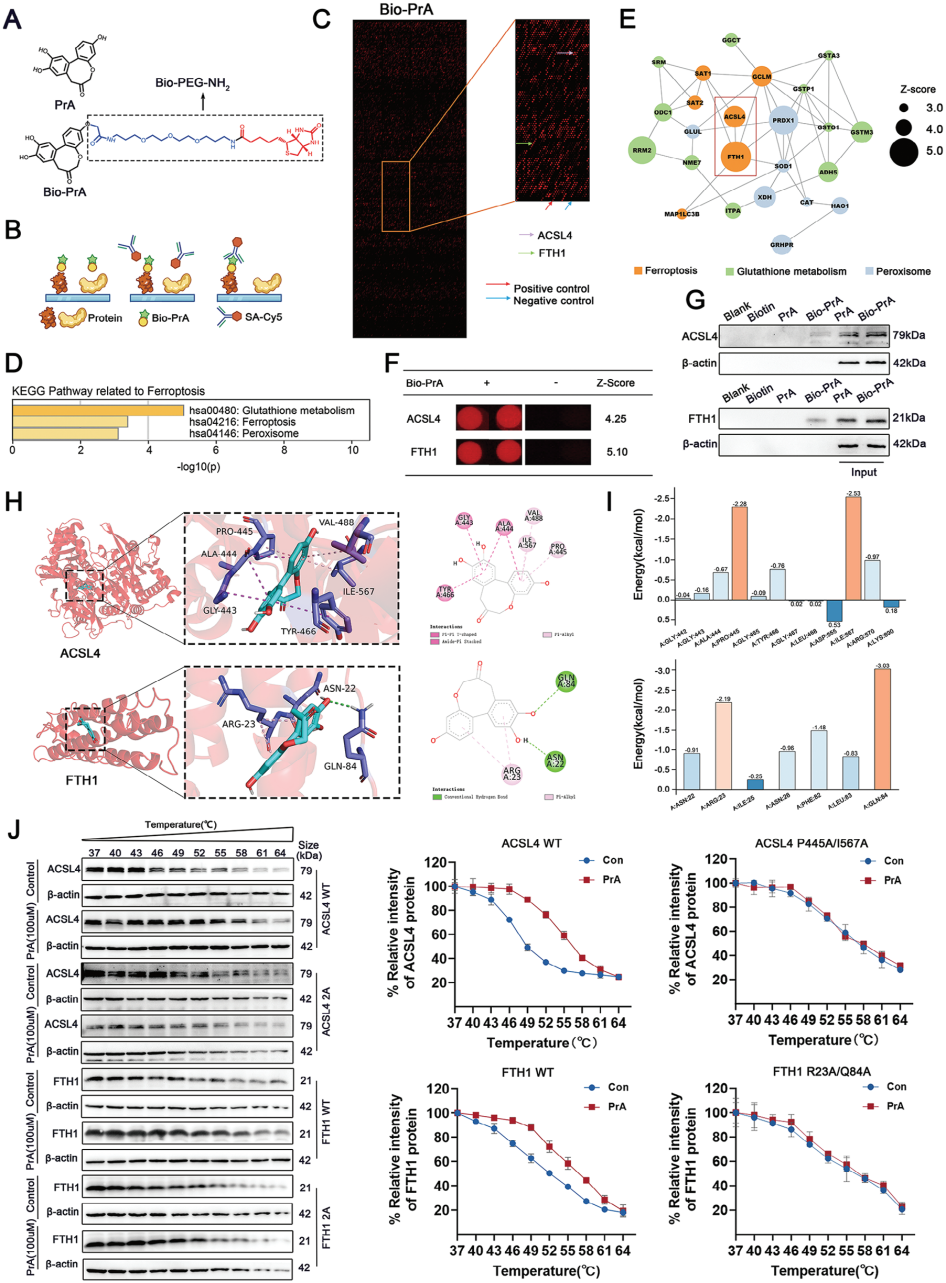
Identification of PrA‐binding proteins on human proteome microarrays. A) Chemical structure of PrA and Bio‐PrA. B) Schematic of the procedure for identifying PrA‐binding proteins using microarrays fabricated with recombinant human proteins. C) Representative image of protein array showing positive (red arrow) and negative control (blue arrow) spots, as well as spots for ACSL4 (purple arrow) and FTH1 (green arrow). D) KEGG pathway of PrA‐binding proteins related to ferroptosis. E) Protein–protein interaction (PPI) network of PrA‐binding proteins related to ferroptosis (circle size represents Z‐score value, orange: the pathway of ferroptosis, green: the pathway of glutathione metabolism, blue: the pathway of peroxisome. Z‐scores were defined as the binding affinity of the target proteins to PrA). F) Magnified image of Bio‐PrA binding to ACSL4 and FTH1 spot on the protein array. Z‐score is shown. G) Biotin alone was used as a control. Bio‐PrA was added to streptavidin‐agarose beads and incubated. Lysates prepared from H9c2 cells were added to the streptavidin‐agarose beads with Bio‐Cel, and the eluent was collected for Western Blot analysis. Total lysates were used as an input control. H) Molecular docking performed the binding pose details between PrA and ACSL4 or FTH1. The figure shows that PrA forms π–π conjugated bonds with ACSL4 Gly‐443, Ala‐444, Tyr‐466, and π–alkyl bonds with ACSL4 Pro‐445, Val‐488, Ile‐567, while PrA forms π–alkyl bonds with FTH1 Arg‐23, and conventional hydrogen bonds with FTH1 Asn‐22, Gln‐84. I) Histograms of the per‐residue energy contributions of key residues involved in Protosappanin A combining with ACSL4 (top panel) and FTH1 (bottom panel). J) Cellular thermal shift assay between PrA and ACSL4 or FTH1. HEK‐293 cells were transfected with plasmids of pcDNA3.1‐3×Flag‐h‐ACSL4 (P445A, I567A) −3×Flag‐zsgreen‐puro or pcDNA3.1‐3×Flag‐h‐FTH1/FTH1 (R23A, Q84A)−3×Flag‐zsgreen‐puro. The curve was fitted using GraphPad Prism 9.0 (*n* = 3 per group). Abbreviations: PrA, protosappanin A.

To determine the underlying mechanism by which PrA inhibits DOX‐mediated cardiomyocyte ferroptosis, biotin‐labeled PrA (Bio‐PrA; **Figure** **4**A) was used in tandem with Cy5‐conjugated streptavidin (Cy5‐SA) to identify the potential target of PrA by detecting the binding of PrA to recombinant proteins fabricated on the HuProt human protein microarray (Figure 4B,C and Figure S5A, Supporting Information). KEGG analysis showed that the ferroptosis‐related pathway was enriched in the PrA‐binding protein sets (Figure 4D). Considering the vital role of PrA in DOX‐mediated ferroptosis, the top two proteins (ACSL4 and FTH1) involved in the ferroptosis pathway, sorted from the Z‐scores, were selected for further analysis (Figure 4E,F).

To validate whether PrA binds to ACSL4 and FTH1 in cell lysates, Bio‐PrA was applied to streptavidin‐agarose beads and co‐incubated with lysates of H9c2 cells. As pull‐down assays indicated, Bio‐PrA bound to ACSL4 and FTH1 proteins in lysates from H9c2 cells (Figure 4G). Subsequently, we performed molecular docking and dynamics simulations to investigate how PrA interacts with ACSL4 and FTH1. As the docking result showed, PrA broadly formed hydrophobic interaction with ACSL4 via π–π and π–alkyl conjugation and exhibited hydrophilic‐lipophilic synergy with FTH1 via conventional hydrogen bonds and π‐alkyl. All of these interactions were beneficial for stabilizing the binding complexes (Figure 4H). As dynamics simulations indicated, the critical residues between PrA and ACSL4 with the top two average energy values were Ile567 and Pro445, and the vital residues between PrA and FTH1 with the top two average energy values were Gln84 and Arg23 (Figure 4I). To validate the molecular docking and dynamics simulation results, we performed a cellular thermal shift assay (CETSA). As shown in Figure 4J, PrA physically interacted with ACSL4 and FTH1, whereas the interactions between PrA and ACSL4 or FTH1 were visibly impaired after mutation of Ile567/Pro445 of ACSL4 and Gln84/Arg23 of FTH1. Taken together, PrA is directly binding to ACSL4 and FTH1.

### 2.5 PrA Inhibits DOX‐Induced Ferroptosis by Preventing ACSL4 Phosphorylation and Activation

**Figure 5:**
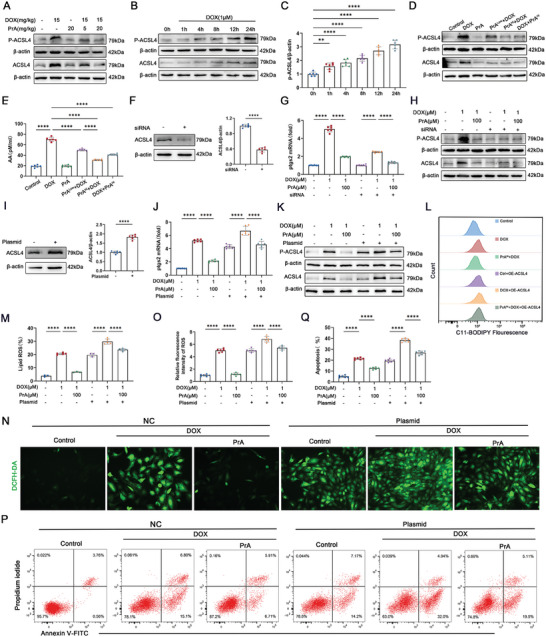
PrA blocks DOX‐induced ACSL4 activation in cardiomyocytes and reduces ROS production and lipid peroxidation. A) Western blot analysis of ACSL4 and p‐ACSL4 (Thr 328) in hearts from control mice and DOX‐treated (20 mg kg^−1^, i.p.) mice with or without PrA (5 or 20 mg kg^−1^, i.g.). B,C) H9c2 cells were exposed to 1 × 10^−6^ m DOX for different durations. The total proteins were extracted and measured for ACSL4 and p‐ACSL4 (Thr 328) levels (*n* = 6 per group). D,E) H9c2 cells were pretreated with PrA in different concentrations for 6 h and then stimulated with 1 × 10^−6^ m DOX for 24 h, or H9c2 cells were challenged with 1 × 10^−6^ m DOX for 24 h and then treated with 100 × 10^−6^ m PrA for 6 h. DMSO was used as vehicle control. Whole‐cell lysates were used for the follow‐up experiment (*n* = 6 per group). D) Western blot was used to determine the levels of ACSL4 and ACSL4 Thr 328 phosphorylation. E) ELISA quantified cellular supernatant AA levels. F–H) H9c2 cells were transfected with siRNA against ACSL4 and negative control siRNA (as the control group). F) Western blot was used to detect knockdown efficiency. Quantification is shown on the right (*n* = 6 per group). G) Relative mRNA levels of Ptgs2 following ACSL4 knockdown (*n* = 6 per group). H) Western blot analysis of ACSL4 and p‐ACSL4 (Thr 328) following ACSL4 knockdown. I–Q) H9c2 cells were transfected with cDNA plasmids encoding ACSL4 or empty vector (negative control, NC). I) Western blot was used to determine ACSL4 expression. Quantification is shown on the right (*n* = 6 per group). J) Effect of ACSL4 overexpression on Ptgs2 levels induction by DOX (*n* = 6 per group). K) Western blot analysis of ACSL4 and p‐ACSL4 (Thr 328) following ACSL4 overexpression (*n* = 6 per group). L,M) Lipid ROS generation and quantitative analysis of ACSL4‐overexpressing H9c2 cells by C11‐BODIPY staining coupled with flow cytometry (*n* = 5 per group). N,O) Representative DCFH‐DA staining images and quantitative analysis of fluorescence intensity for ACSL4‐overexpressing H9c2 cells (green, scale bar: 100 µm, *n* = 6 per group). P,Q) Flow cytometry and quantitative analysis of Annexin V‐APC and PI in ACSL4‐overexpressing H9c2 cells (*n* = 6 per group). C,F,G,I,J,O) Some of the data was normalized. Summary data are presented as the mean ± SEM. Statistical significance was determined using one‐way ANOVA with D,F,I,L,N,P) Tukey's multiple comparisons tests and E,H) multiple unpaired 2‐tailed Student *t*‐tests. *p < 0.05, **p < 0.01, ***p < 0.001, ****p < 0.0001. Abbreviations: DOX, doxorubicin; i.p., intraperitoneal; i.g., intragastric; PrA, protosappanin A.

ACSL4 is an isozyme responsible for metabolizing polyunsaturated fatty acid (PUFA) metabolism. Recent evidence indicates that Thr328 phosphorylation is essential for ACSL4 enzymatic activation to enhance ferroptosis sensitivity.^[^ ^15^ ^]^ Considering that PrA directly binds to ACSL4 and inhibits DOX‐induced ferroptosis, we investigated whether PrA protects against DOX‐induced ferroptosis by interfering with ACSL4's enzymic activation. Western blotting showed that ACSL4 phosphorylation and protein levels were both upregulated in the heart tissues of DOX‐treated mice, and these changes were reversed by PrA treatment in a dose‐dependent manner (**Figure** **5**A and Figure S6A,B, Supporting Information). ACSL4 protein and Thr328 phosphorylation levels were gradually increased in DOX‐conditioned H9c2 cells over time (Figure 5B,C and Figure S6C, Supporting Information). The ACSL4 protein and Thr328 phosphorylation levels were also suppressed in high‐dose PrA‐treated H9c2 cells after DOX exposure for 24 h. Notably, pretreatment with PrA also reduced ACSL4 protein levels and Thr328 phosphorylation in DOX‐conditioned H9c2 cells (Figure 5D and Figure S6D,E, Supporting Information). Furthermore, PrA treatment alone did not affect ACSL4 protein or Thr328 phosphorylation levels in murine heart tissue or H9c2 cells derived from the control group. Thus, the binding of PrA to ACSL4 disturbs ACSL4 phosphorylation at Thr328 and its protein expression in the presence of DOX.

ACSL4‐mediated PUFA metabolism facilitates the synthesis of arachidonic acid (AA)‐containing phospholipids and lipid peroxidation, ultimately driving ferroptosis.^[^ ^16^ ^]^ Consequently, we measured the AA levels in H9c2 cells treated with or without PrA in the presence of DOX. ELISA assay confirmed that PrA pretreatment alone did not change AA production; however, it reversed DOX‐mediated AA generation from the cellular supernatant in H9c2 cells. Despite post‐DOX exposure, a high dose of PrA retained its capacity to rescue AA accumulation caused by DOX (Figure 5E). Based on these results, we concluded that PrA binding to ACSL4 inhibits DOX‐induced ACSL4 phosphorylation and enzymatic activity, further disrupting the downstream metabolism of PUFAs and lipid peroxidation.

To confirm whether PrA inhibited DOX‐induced ferroptosis by preventing ACSL4 phosphorylation and activation, Ptgs2 mRNA levels were evaluated in H9c2 cells transfected with siRNA or an overexpressing plasmid specifically targeting ACSL4 under the aforementioned conditions. In agreement with the data of PrA administration, silencing ACSL4 (Figure 5F) suppressed Ptgs2 mRNA levels in DOX‐conditioned H9c2 cells, indicating the inhibitory effect of ACSL4 knockdown on DOX‐induced cardiomyocyte ferroptosis (Figure 5G). Moreover, the inhibitory effect of ACSL4‐siRNA and PrA on DOX‐induced ferroptosis was associated with reduced ACSL4 Thr328 phosphorylation (Figure 5H and Figure S6F,G, Supporting Information). In contrast, ACSL4 overexpression (Figure 5I) promoted Ptgs2 mRNA expression (Figure 5J) and ACSL4 Thr328 phosphorylation levels (Figure 5K and Figure S6H, Supporting Information) in H9c2 cells with DOX exposure. Under ACSL4‐saturated conditions, PrA had a reduced salvage capacity on DOX‐mediated enlargement of Ptgs2 mRNA (Figure 5J), ACSL4 protein and Thr328 phosphorylation (Figure 5K and Figure S6H,I, Supporting Information), and lipid and intracellular ROS levels (Figure 5L–O) in H9c2 cells. Given these findings, flow cytometry was conducted, revealing that ACSL4 overexpression impaired the protective effect of PrA against DOX‐induced cardiomyocyte death (Figure 5P,Q). Taken together, our data indicated that PrA inhibits cardiomyocyte ferroptosis by preventing DOX‐induced ACSL4 phosphorylation at Thr328 and its subsequent activation.

### 2.6 PrA Protects against DOX‐Mediated Ferroptosis by Reducing Autophagic Degradation of FTH1 in Lysosomal

**Figure 6:**
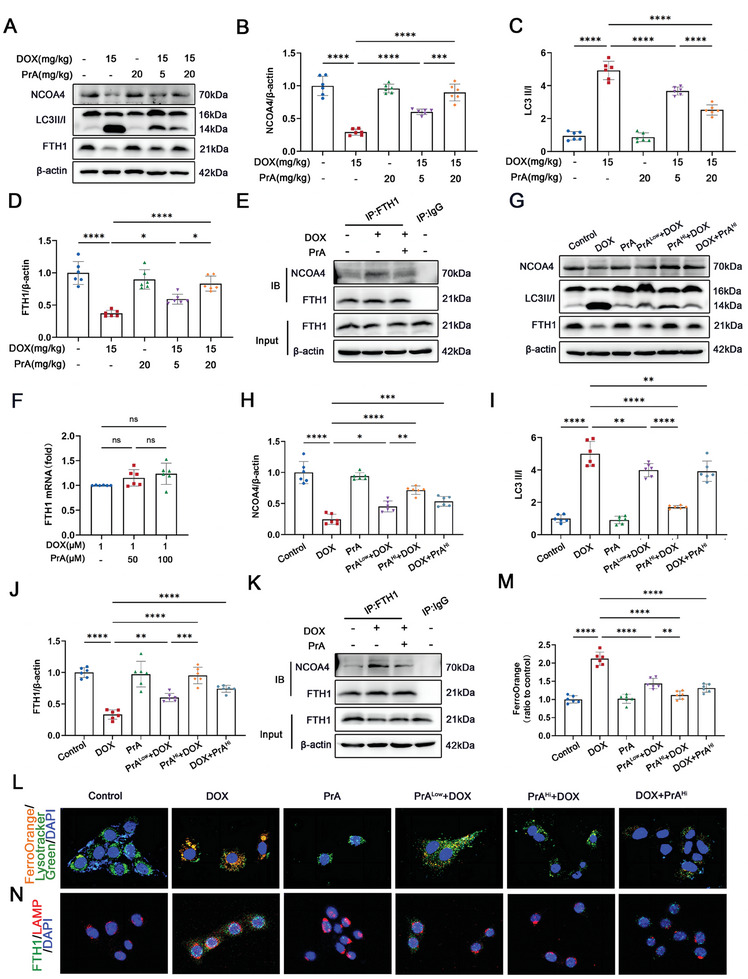
PrA inhibits FTH1's autophagic degradation in lysosome. A–D) Western blot and quantitative analysis of cardiac NCOA4, LC3 II/I and FTH1 protein levels in control mice and mice treated with DOX with or without PrA (*n* = 6 per group). E) NCOA4‐FTH1 interactions were analyzed by coimmunoprecipitation in control mice and mice treated with DOX with or without PrA (*n* = 3 per group). F) Relative mRNA levels of FTH1 were measured in control H9c2 cells and cells treated with PrA in different concentrations for 6 h and then stimulated with 1 × 10^−6^ m DOX for 24 h (*n* = 6 per group). G) Western blot analysis of NCOA4, LC3 II/I and FTH1 in control H9c2 cells and cells pretreated with PrA in different concentrations for 6 h and then stimulated with 1 × 10^−6^ m DOX or 24 h or H9c2 cells were challenged with 1 × 10^−6^ m DOX for 24 h and then treated with 100 × 10^−6^ m PrA for 6 h. H–J) Quantitative analysis of the protein levels of H) NCOA4, I) LC3 II/I, and J) FTH1 in panel G (*n* = 6 per group). K) NCOA4‐FTH1 interactions were analyzed by coimmunoprecipitation in control H9c2 cells and cells treated with DOX with or without PrA (*n* = 3 per group). L,M) Representative confocal images and quantitative analysis of FerroOrange co‐localized with LysoTracker Green in H9c2 cells (orange: FerroOrange, green: LysoTracker Green, blue: DAPI, scale bars:20 µm, *n* = 6 per group). N) Representative immunofluorescence imaging of FTH1in H9c2 cells. (green: FTH1, red: LAMP, blue: DAPI, scale bars: 20 µm). B–D,F,H–J,M) Some of the data was normalized. Summary data are presented as the mean ± SEM. Statistical significance was determined using one‐way ANOVA with Tukey's multiple comparisons test. *p < 0.05, **p < 0.01, ***p < 0.001, ****p < 0.0001. Abbreviations: DOX, doxorubicin; PrA, protosappanin A; SEM, standard error of mean.

The key ferritin subunit, FTH1, has been reported to mediate ferritinophagy by binding to the cargo receptor NCOA4 and transferring the iron‐containing ferritin complex to the autolysosome for autophagic degradation, resulting in the release of free Fe^2+^ and increased cell susceptibility to ferroptosis.^[^ ^17^ ^]^ Our data showed that PrA could directly bind to FTH1, and restore the DOX‐induced alterations in FTH1 protein levels (Figure 3F) and intracellular Fe^2+^ production (Figure 3J) in H9c2 cells. Thus, we speculated that PrA might disrupt the DOX‐mediated NCOA4‐FTH1 interaction and subsequently disturb ferritinophagy by binding to FTH1. To validate this possibility, we first analyzed the protein expression of FTH1, NCOA4, and the autophagic marker LC3‐II/I in DIC murine hearts treated with or without PrA, using western blotting. Compared to control mice, DOX‐treated mice exhibited lower FTH1 and NCOA4 expression as well as a higher LC3‐II/I percentage in cardiac tissues, whereas PrA intervention reversed these changes (**Figure** **6**A–D). Co‐immunoprecipitation assays further showed that PrA administration weakened the interaction between FTH1 and NCOA4 in the DOX‐treated mice (Figure 6E). In agreement with the in vivo results, PrA restored FTH1 protein levels instead of mRNA expression caused by DOX in H9c2 cells (Figure 6F), suggesting that PrA regulates FTH1 protein levels in the presence of DOX by inhibiting protein degradation rather than by upregulating gene transcription. Similarly, PrA rescued the protein expression of NCOA4 and the LC3‐II/I percentage, as well as the NCOA4‐FTH1 interaction in H9c2 cells, both before and after DOX exposure, while PrA treatment alone had no impact on these indices (Figure 6G–K). Therefore, our data support the conclusion that the combination of PrA and FTH1 inhibits DOX‐triggered NCOA4‐FTH1 internal interaction and FTH1 protein autophagic degradation.

To elucidate the mechanism underlying the prevention of DOX‐induced ferroautophagy by PrA, we evaluated the intracellular content and subcellular location of Fe^2+^ using specific fluorescent probes for tracing Fe^2+^ and lysosomes. Laser confocal microscopy revealed that DOX‐treated H9c2 cells emitted strong Fe^2+^ fluorescence in lysosomes, which was weakened by increasing doses of PrA administered prior to DOX exposure, indicating that PrA inhibited the DOX‐induced release of intracellular Fe^2+^ from lysosomes into the cytoplasm (Figure 6L,M). According to the immunofluorescence assay, DOX treatment accelerated the translocation of FTH1 to lysosomes. In contrast, the coocalization of FTH1 and lysosomes was abrogated under the PrA challenge (Figure 6N). Despite post‐DOX treatment, high‐dose PrA still inhibited the colocalization of FTH1 and lysosomes, as well as the release of Fe^2+^ into the cytoplasm (Figure 6L–N). Collectively, the combination of PrA and FTH1 disrupted the NCOA4‐FTH1 interaction, inhibiting ferritinophagy and the release of Fe^2+^ from lysosomes, thereby improving DOX‐induced cardiomyocyte ferroptosis.

### 2.7 PrA‐Mediated Inhibition of Ferroptosis Attenuates Ischemia‐Reperfusion ‐Induced Cardiac Injury and Dysfunction

**Figure 7:**
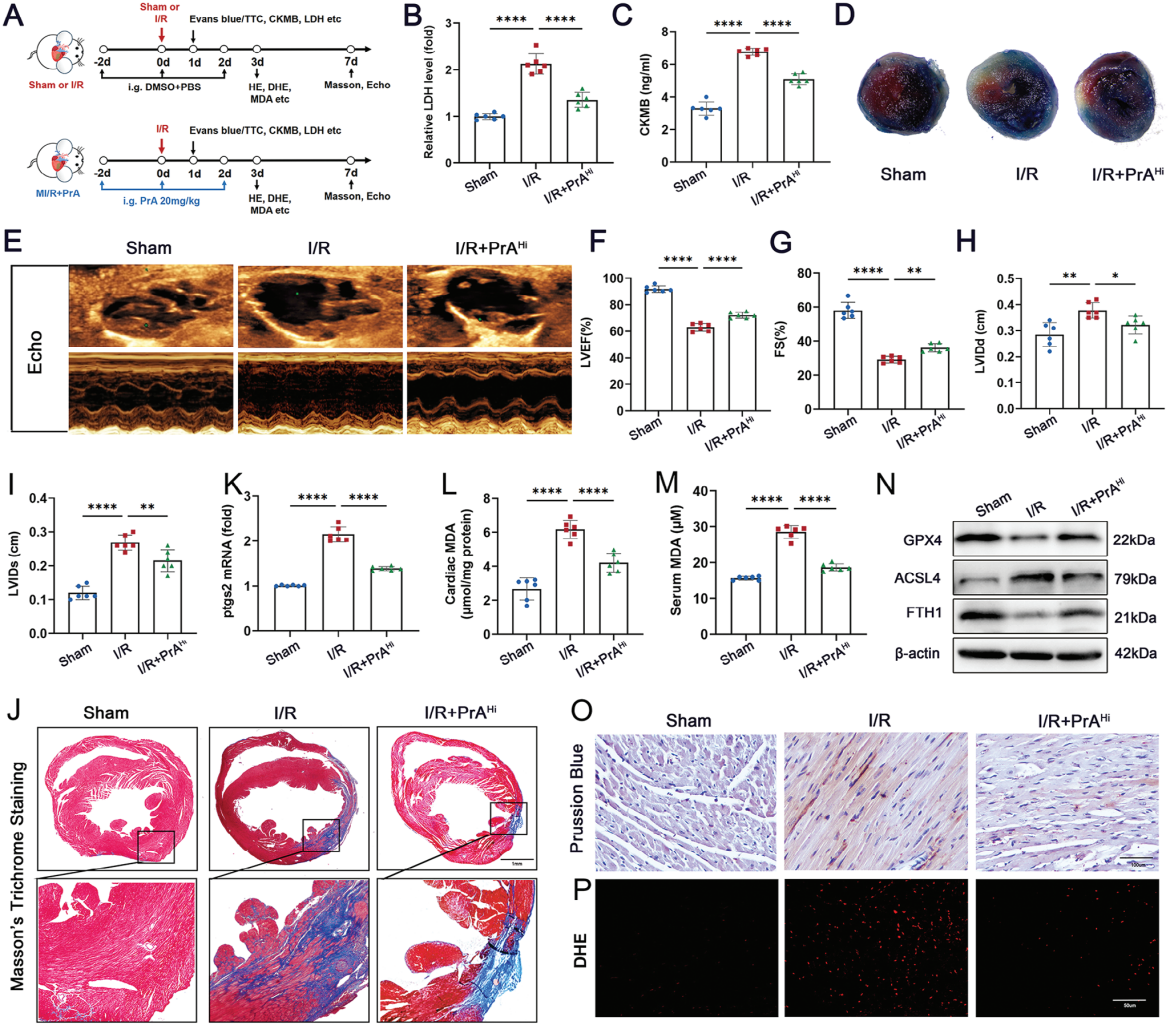
PrA ameliorates ischemia/reperfusion (I/R)‐induced cardiac injury and ferroptosis. A) Schematic diagram of animal experiment process. Time points in the image are representative. B) Serum LDH and C) CK‐MB (fivefold dilution) levels were measured in mice 24 h after sham or I/R surgery, treated with or without PrA (20 mg kg^−1^ i.g.) (*n* = 6 per group). D) Evans blue/triphenyltetrazolium chloride (TTC) staining of heart sections in mice 24 h after sham or I/R surgery, treated with or without PrA (20 mg kg^−1^ i.g.) (scale bars:100 µm). E–I) Representative echocardiographic images from mice 7 d after sham or I/R surgery, treated with or without PrA (20 mg kg^−1^ i.g.). F–I) Quantitative analysis of LVEF, FS, LVIDd, and LVIDs (*n* = 6 per group). J) Representative images of Masson's trichrome staining in mice 7 d after sham or I/R surgery, treated with or without PrA (20 mg kg^−1^ i.g.) (scale bars:1 mm, *n* = 6 per group). K) Relative mRNA levels of Ptgs2 (*n* = 6 per group). Quantitative analysis of L) cardiac MDA and (M) serum MDA (*n* = 6 per group). N) Western blots of cardiac GPX4, ACSL4, and FTH1 (*n* = 6 per group). O,P) Representative images of Prussian blue iron staining with DAB enhancement (O, scale bar:100 µm) and fluorescent immunohistochemistry staining for DHE (P, scale bar: 20 µm). B,K) Some of the data was normalized. Summary data are presented as the mean ± SEM. Statistical significance was determined using one‐way ANOVA with Tukey's multiple comparisons test. *p < 0.05, **p < 0.01, ***p < 0.001, ****p < 0.0001. Abbreviations: CK‐MB,  creatine kinase‐MB; LDH, lactate dehydrogenase; i.g., intragastric; PrA, protosappanin A.

Recent studies have indicated a strong correlation between ferroptosis and myocardial I/R, suggesting that ferroptosis intervention may offer therapeutic benefits in enhancing cardiac function post‐reperfusion.^[^ ^12^ ^]^ Therefore, we tested whether PrA treatment exerts a therapeutic effect on myocardial I/R injury. Myocardial I/R murine models were established and treated with or without PrA (20 mg kg^−1^) for indicated days (**Figure** **7**A). PrA treatment partially recovered the I/R‐induced elevation of serum myocardial injury markers activity, including CK‐MB and LDH, compared to I/R mice (Figure 7B,C). As shown in Figure 7D and Figure S7A (Supporting Informaton), the infarct size was significantly lower in the PrA‐pretreated group than in the I/R group. Subsequently, we evaluated cardiac function using echocardiography. I/R‐induced cardiac dysfunction was primarily manifested by changes in LVEF, FS, LVIDd, and LVIDs, which resumed after PrA treatment (Figure 7E–I). Furthermore, histological staining revealed that PrA significantly ameliorated the I/R‐induced cardiac fibrosis (Figure 7J).

To clarify the protective mechanism of PrA against ferroptosis in myocardial I/R injury, we assessed the mRNA levels of Ptsg2 in I/R mice hearts using PCR. As expected, the Ptgs2 mRNA levels were significantly downregulated in PrA‐treated I/R mice (Figure 7K). In addition, the cardiac and serum MDA levels in the I/R group were higher than those in the PrA treatment group (Figure 7L,M). PrA treatment restored the I/R‐induced changes in GPX4, ACSL4, and FTH1 proteins (Figure 7N and Figure S7B–D, Supporting Informaton), iron (Figure 7O and Figure S7E, (Supporting Informaton)), and ROS content (Figure 7P and Figure S7F, Supporting Informaton) in mouse cardiac tissue. Our results suggest that PrA has antiferroptotic and cardioprotective activities against myocardial I/R injury.

## 3 Discussion

At present, there is no consensus on the prevention and treatment of cardiovascular toxic effects induced by chemotherapy drugs. Existing strategies to alleviate cardiotoxicity mainly involve adjusting drug delivery patterns and dosage form design, or administering cardioprotective medicine,^[^ ^8^, ^18^ ^]^ while their clinical application and development are generally limited due to increased side effects, poor in vivo activity, and high costs. Consequently, there is a need to explore practical interventional approaches to mitigate chemotherapy‐induced cardiotoxicity. Our study is the first to demonstrate that PrA protects against DOX‐induced cardiac injury and dysfunction in a dose‐dependent manner in a murine DIC model. Furthermore, PrA‐mediated protective efficacy at 20 mg kg^−1^ was partially superior to that of DXZ in terms of improving DOX‐induced myocardial damage, showing its potential practical and clinical application value in the treatment of DIC.

Cardiomyocyte death is a pathological feature of DOX‐induced myocardial damage and cardiac dysfunction.^[^ ^19^ ^]^ The emerging form of cell death, ferroptosis, is one of the principal pathogenic mechanisms of cancer,^[^ ^20^ ^]^ ischemic organ damage,^[^ ^21^ ^]^ and other degenerative diseases closely related to lipid peroxidation.^[^ ^22^ ^]^ Substantial evidence indicates that inhibition of cardiomyocyte ferroptosis is a promising preventative therapeutic strategy for DIC.^[^ ^7^, ^23^ ^]^ However, it remains unclear whether PrA regulates DOX‐mediated ferroptosis in cardiomyocytes. Recent studies have revealed that ferroptosis is primarily induced by exogenous (transporter‐dependent) and endogenous (enzyme‐regulated) pathways.^[^ ^24^ ^]^ In addition, imbalances in lipid metabolism, iron overload, and oxidative stress have been identified as precipitating factors for ferroptosis.^[^ ^25^ ^]^ Building on this background, our research demonstrated that PrA could alleviate DOX‐induced ferroptosis in heart tissue and cardiomyocytes via the aforementioned endogenous and exogenous pathways by inversing DOX‐induced changes in GPX4, FTH1, ACSL4, Fe^2+^, lipid peroxidation products, and ROS levels.

Accumulating evidence suggests a strong link between ferroptosis and mitochondrial signaling.^[^ ^26^ ^]^ Cellular ferroptosis results in structural and functional irregularities in the mitochondria, further exacerbating cell susceptibility to ferroptosis and forming a positive feedback loop.^[^ ^27^ ^]^ Studies have shown that mitochondrial disorders drive ferroptosis via ROS generation, glutamine catabolism, and iron metabolism.^[^ ^28^ ^]^ Mitochondria with complete functions acquire the ability to resist ferroptosis mainly by promoting fatty acid beta‐oxidation and inhibiting GPX4 catalytic activity and lipid peroxidation.^[^ ^29^ ^]^ In another recent study, the cardiac cGAS‐STING pathway regulated by DOX initiated mitochondrial dysfunction, which is an indispensable mechanism for myocardial injury and cardiac dysfunction.^[^ ^30^ ^]^ In support of these findings, we observed that PrA ameliorated DOX‐induced cardiomyocyte ferroptosis in a mitochondria‐dependent manner by monitoring the related indices for ferroptosis, mitochondrial morphology, and function.

ACSL4, a crucial isozyme in the endogenous pathway driving ferroptosis, can synergistically promote the incorporation of PUFA into phospholipids to form PUFA‐containing phospholipids, which are susceptible to lipoxygenase‐initiated oxidation reactions, destroying the lipid bilayer and impairing membrane function.^[^ ^31^ ^]^ The proferroptotic and potential pathogenic effects of ACSL4 have recently been mapped to various multisystem diseases, including radiation‐induced intestinal injury,^[^ ^32^ ^]^ cerebral ischemia/reperfusion,^[^ ^33^ ^]^ and cardiac microvascular injury.^[^ ^34^ ^]^ Despite the abundant evidence mentioned above, little research has been conducted on the mechanism of action of ACSL4 in DIC. Studies have reported that ACSL4 protein is upregulated in cardiomyocytes during DOX‐induced ferroptosis.^[^ ^35^ ^]^ However, the mechanism by which DOX regulates ACSL4 in DIC remains unknown. A recent study showed that Thr328 at ACSL4 is phosphorylated by PKCβII to form a dimer complex, further activating its enzymatic action to drive lipid peroxidation and ferroptosis.^[^ ^14^ ^]^ By screening potential downstream target proteins that bind to PrA, we found that PrA physically binds to ACSL4 mainly via the Ile567/Pro445 residues, indicating that PrA protects against DOX‐induced cardiotoxicity by directly targeting the ferroptosis pathway. Combined with the results of PrA inhibition of ACSL4 protein expression under DOX‐challenged conditions, we speculated that PrA inhibits DOX‐induced cardiomyocyte ferroptosis by disturbing ACSL4's enzymatic activity. Based on a series of in vivo and in vitro experiments, we demonstrated for the first time that DOX exposure upregulates ACSL4 phosphorylation and protein expression in cardiomyocytes and heart tissues, activating its enzymatic reaction, followed by lipid peroxidation product aggregation and increased susceptibility to ferroptosis. In contrast, PrA effectively restored DOX‐mediated ferroptosis, both before and after DOX treatment, suggesting that PrA alleviated DOX‐induced ferroptosis by binding to ACSL4 and inhibiting its enzymatic activity.

Iron transport and overload are fundamental mechanisms that exogenously drive ferroptosis.^[^ ^24^ ^]^ Under normal conditions, iron is stored in ferritin (composed of the ferritin light chain and FTH1), and ferroportin maintains the iron balance by promoting the cellular uptake of iron. In the presence of intracellular iron deficiency, ferritin combines with the autophagy receptor to form a complex, further linking to LC3‐II and transportation to autophagosomes for degradation, allowing the release of ferritin‐bound iron into the cytoplasm, a process referred to as ferroautophagy.^[^ ^36^ ^]^ Research has shown that the cytoplasmic autophagy receptor NCOA4 selectively recognizes the FTH1 subunit in ferritin and that FTH1 R23 is an influential structure essential for ferritin binding to NCOA4.^[^ ^16^ ^]^ Interference with the interaction between FTH1 and NCOA4 prevents iron autophagy and further reduces mitochondrial ferric content, conferring a critical therapeutic role in pulmonary fibrosis,^[^ ^37^ ^]^ glaucoma,^[^ ^38^ ^]^ and neurodegenerative diseases.^[^ ^39^ ^]^ In our research, FTH1 was identified as a downstream binding protein of PrA using molecular docking and thermal shift experiments. Based on these findings, we explored the mechanisms of interaction between PrA and FTH1 in DOX‐induced ferroptosis. In our in vivo and in vitro experiments, PrA rescued DOX‐mediated alterations in FTH1, NCOA4, and LC3‐II/I protein levels and FTH1‐NCOA4 interaction in heart tissues and cardiomyocytes. Combined PCR and western blot analyses showed that PrA regulates DOX‐conditioned FTH1 expression at the post‐transcriptional level, indicating that PrA may regulate FTH1‐mediated ferroautophagy in the presence of DOX. This assumption was further validated using a specific fluorescence probe to trace intracellular Fe^2+^ and lysosomes. Immunofluorescence and confocal microscopy showed that PrA prevented FTH1‐lysosome co‐localization and the release of Fe^2+^ into the cytoplasm in the presence of DOX. Therefore, we conclude that PrA binds to FTH1, competitively inhibits FTH1‐NCOA4 interaction, blocks FTH1‐mediated ferroautophagy, and protects cardiomyocytes from ferroptosis.

Recent evidence has revealed that ferroptosis is a pivotal etiological factor of myocardial I/R injury, and inhibiting ferroptosis effectively reduces myocardial cell death and improves left ventricular systolic function caused by I/R.^[^ ^12b^ ^]^ To investigate the possibility of using PrA as a ferroptosis inhibitor in clinical applications and translational development, we analyzed the effect of PrA on myocardial I/R injury. Remarkably, our results confirmed the cardioprotective and antiferroptotic effects of PrA in a mouse model of myocardial I/R. In follow‐up experiments, we will further validate the specific mechanism by which PrA regulates ferroptosis in AC16 cardiomyocytes. Moreover, it is worth discovering the therapeutic role and antiferroptotic activity of PrA in other ferroptosis‐mediated diseases. Additionally, we intend to conduct a series of clinical trials to validate the safety and efficacy of PrA for the treatment of antineoplastic drug‐mediated cardiotoxicity.

## 4 Conclusion

Our study is the first to reveal that PrA alleviates DOX‐induced myocardial damage by inhibiting the ACSL4/FTH1‐dependent ferroptosis pathway. Furthermore, the cardioprotective and antiferroptotic effects of PrA were elucidated in myocardial I/R murine models. Collectively, our data suggest that PrA could serve as a novel dual ferroptosis‐targeting inhibitor with promising advances and transformation value for potential therapeutic avenues in DIC and other ferroptosis‐mediated disorders.

## 5 Experimental Section

### Drugs

PrA (JOT‐10793, purity≥98%) was purchased from Chengdu Pufei De Biotech Co., Ltd. (Chengdu, China). Doxorubicin (DOX, D107159, purity≥98%) was purchased from Aladdin (Shanghai, China). Dexrazoxane hydrochloride (DXZ, ab141109, purity≥98%) was purchased from Abcam (Shanghai, China). The antibodies used in this study are listed in Table S2 (Supporting Information).

### Mice

Wild‐type C57BL/6 mice (6–8 weeks old, 20 ± 2 g) were purchased from the Beijing Lab Animal Research Center (Beijing, China). Mice were housed in a temperature‐controlled environment (22–24 °C) with a 12 h light‐dark cycle and fed a standard rodent laboratory diet. All experimental methods and protocols were approved by the Animal Care and Use Committee of the Second Affiliated Hospital of Harbin Medical University (No. Sydwgzr2020‐014) following the ARRIVE guidelines. Given that C57BL/6 male mice are commonly used to establish the DIC models,^[^ ^40^ ^]^ male animals were primarily used in this study. However, considering significant gender differences in DOX‐induced cardiotoxicity,^[^ ^41^ ^]^ female C57BL/6 mice were further included for validation.

### Doxorubicin‐Induced Acute Cardiac Injury Model

For acute experiments, 6–8 weeks old mice received a single intraperitoneal (i.p.) injection of DOX (15 mg kg^−1^ body weight dissolved in sterile saline)^[^ ^42^ ^]^ or saline. Where indicated, mice received pretreatment of PrA (5 mg kg^−1^ or 20 mg kg^−1^)^[^ ^11^ ^]^ via oral gavage or DXZ (25 mg kg^−1^)^[^ ^43^ ^]^ via i.p. injection 48 h before DOX treatment, and then continuously received the same treatment for two more times 2 and 48 h after DOX treatment, with the same volume of vehicle (Veh; 10% dimethylsulfoxide [DMSO] with phosphate buffer saline [PBS]) intraperitoneally administered as the control. PrA and DXZ were dissolved in 10% DMSO and diluted in sterile saline.

### I/R Myocardial Injury Model

Adult male mice (6–8 weeks old) were anesthetized with tribromoethanol (M2910, AiBei Biotechnology, Nanjing, China, 20 mL kg^−1^, i.p.) and placed in a supine position on the experimental board. Following sedation, the mice were intubated using a small‐animal ventilator. After a left thoracotomy incision was made, ischemia was induced by reversible ligation of the left anterior descending (LAD) coronary artery using a 7‐0 silk suture with a slipknot. Proper ligation was confirmed by the visual observation of the left ventricular wall turning pale. After 30 min of regional ischemia, the slipknot was released, leading to the recovery of the discolored myocardium distal to the ligation. PrA (20 mg kg^−1^)^[^ ^11^ ^]^ was administered by oral gavage 24 and 2 h before surgery. Sham‐operated control mice (sham MI/R) underwent the same surgical procedure but without ligation of the LAD coronary artery.

### Cell Culture

Neonatal primary cardiomyocytes were isolated as previously described.^[^ ^44^ ^]^ H9c2 and HEK‐293 cells were obtained from the Cell Bank of the Chinese Academy of Sciences (Shanghai, China). The cells were cultured in Dulbecco's modified Eagle's medium (DMEM, Gibco) supplemented with 10% fetal bovine serum (FBS, Pricella) and 1× penicillin‐streptomycin (Beyotime Biotechnology, Shanghai, China) and incubated in a humidified atmosphere consisting of 5% CO2 and 95% air at 37 °C. DOX was applied to H9c2 cells and primary cardiomyocytes for 24 h^[^ ^45^ ^]^ at the indicated concentrations. PrA interventions, at the indicated concentrations,^[^ ^11^ ^]^ were applied 6 h before or after DOX treatment.

### Proteome Microarray Assays

Identification of PrA‐interacting proteins was provided by Wayen Biotechnology (Shanghai, China) using a HuProt 20 K^[^ ^46^ ^]^ Proteome microarray containing 23145 purified human proteins. Briefly, the microarrays were immersed in a blocking buffer (5% bovine serum albumin in PBS‐T) at room temperature (RT, 20 °C) for 1 h. The microarrays were then incubated with 10 × 10^−6^ m biotinylated PrA or free biotin for 1 h. After washing, the proteome microarrays were placed in 0.1% Cy5‐streptavidin solution at RT in the dark for 20 min. After washing again and centrifuging (1000×*g*, 2 min) to dry, observations and data analysis were performed using a GenePix 4000B microarray scanner (Axon Instruments, USA) and GenePix TM Pro v6.0 software (Axon Instruments, USA). The protein spot with a Z‐Score (signal strength indicator) ≥ 2.8 in a PrA‐Bio‐treated microarray and a Z‐Score < 2.8 in a biotin‐treated microarray was identified as the candidate positive protein. The Z‐Score ratio of PrA‐Bio‐treated versus biotin‐treated was defined as IMean‐Ratio, and the candidate positive protein with an IMean‐Ratio ≥1.4 was identified as the final PrA target protein.

### Statistical Analysis

Data were analyzed and plotted using GraphPad Prism software (version 9.0, GraphPad Software, Boston, MA, USA). The biological replicate (*n*) for each statistical analysis is shown in the figure legends. Survival curves were constructed using Kaplan–Meier analysis with the log‐rank (Mantel‐Cox) test. All summary data are presented as mean ± standard deviation and were evaluated using a two‐tailed unpaired Student's t‐test between two groups or one‐way ANOVA with Tukey's multiple comparisons between multiple groups. Differences were considered statistically significant at *p* < 0.05.

## Conflict of Interest

The authors declare no conflict of interest.

## Supporting information

Supporting Information

## Data Availability

The data that support the findings of this study are available from the corresponding author upon reasonable request.
