# Supplementary material for: Protosappanin A Protects DOX‐Induced Myocardial Injury and Cardiac Dysfunction by Targeting ACSL4/FTH1 Axis‐Dependent Ferroptosis
Source: Adv Sci (Weinh). 2024 Jul 10;11(34):2310227. doi: 10.1002/advs.202310227 (PMC11425893; doi:10.1002/advs.202310227)
Supplement: Supplementary file 1 — Supporting Information [file ADVS-11-2310227-s001.docx]

**Supplementary Material**

**Protosappanin A Protects DOX-Induced Myocardial Injury and Cardiac Dysfunction by Targeting ACSL4/FTH1 Axis-Dependent Ferroptosis**

*Jingxuan Cui****^#^****, Yujia Chen****^#^****, Qiannan Yang****^#^****, Peng Zhao****^#^****, Mian Yang, Xiaoqi Wang, Ge Mang, Xiangyu Yan, Di Wang, Zhonghua Tong, Penghe Wang, Yingjin Kong, Naixin Wang, Dongni Wang, Nana Dong, Mingyang Liu*, Mingyan E*, Maomao Zhang*, and Bo Yu*

***^#^*** Co-first authors: Jingxuan Cui, Yujia Chen, Qiannan Yang, Peng Zhao

*** Corresponding authors: Maomao Zhang, Mingyan E, Mingyang Liu

E-mail: 100252@hrbmu.edu.cn (Prof. Maomao Zhang),

600368@hrbmu.edu.cn (Mingyan E), 822674@hrbmu.edu.cn (Mingyang Liu)

**CONTENT:**

**Experimental section**

**Supplementary Figure S1.** PrA ameliorates DOX-induced cardiac injury in female mice.

**Supplementary Figure S2.** PrA inhibits DOX-induced cardiac ferroptosis.

**Supplementary Figure S3.** PrA mitigates DOX-induced iron accumulation and lipid peroxidation and improves mitochondrial function.

**Supplementary Figure S4.** PrA protects primary cardiomyocytes against DOX-triggered cellular damage.

**Supplementary Figure S5.** Representative image of protein array without PrA as a control experiment**.**

**Supplementary Figure S6.** PrA inhibits DOX-induced ACSL4 threonine 328 phosphorylation.

**Supplementary Figure S7.** The inhibitory effects of PrA on ischemia/reperfusion (I/R)-induced ferroptosis.

**Supplementary Table S1.** Primer pairs for quantitative real-time PCR.

**Supplementary Table S2.** Antibodies used for immunoblotting and immunofluorescence.

**Experimental section**

**Biomarkers of cardiac injury**

The level of creatine kinase-MB (CK-MB) was measured with ELISA kit (JLC3288, Jingkang Biotechnology, Shanghai, China), according to the manufacturer’s recommendations.

The enzyme activity of lactate dehydrogenase (LDH) was detected using LDH Assay Kit (C0017, Beyotime Biotechnology, Shanghai, China) according to the manufacturer’s instructions.

**Measurement of MDA and GSH content**

Serum and cardiac malondialdehyde (MDA) levels were measured using a Lipid Peroxidation MDA assay kit (S0131, Beyotime, Shanghai, China) in accordance with the manufacturer’s instructions.

Cardiac total glutathione (GSH) levels were measured using a GSH and GSSG Assay kit (S0053, Beyotime, Shanghai, China) according to the manufacturer's protocol.

**Echocardiography**

Transthoracic echocardiography was performed on anesthetized mice using a high-resolution in vivo VIVID E9 imaging system (GE Healthcare, USA). Transthoracic echocardiography at the parasternal long-axis and short-axis views were performed and recorded. Left ventricular (LV) dimensions and wall thicknesses were estimated via parasternal short-axis M-mode images. LV mass and functional parameters, including the percentage of left ventricular ejection fractions (LVEF), fractional shortening (FS), left ventricular internal dimension in diastole (LVIDd), and left ventricular internal dimension in systole (LVIDs) were calculated using the above-mentioned primary measurements and the accompanying software. Doxorubicin (DOX)-induced cardiac morphology was observed at the short-axis, while ischemia/reperfusion (I/R)-induced cardiac morphology was observed at the long-axis.

**Histology**

Mice heart tissues were fixed in 4% paraformaldehyde and further embedded in paraffin according to standard protocols. Sections with 4 µm thickness were stained with Hematoxylin and eosin (H&E, G1120, Solarbio, Beijing, China) for routine histological analysis, and with Sirius red (PH1098, Phygene Scientific, Beijing, China) and Masson’s trichrome (G1340, Solarbio, Beijing, China) to evaluate cardiac collagen deposition and fibrosis, following the manufacturer’s instructions. For each mouse, three adjacent sections were quantified using Image J software to measure the iron level. Sections of the heart were stained with a Prussian blue iron stain kit (Enhance With DAB, G1428, Solarbio, Beijing, China), according to the manufacturer’s instructions.

For determination of cardiac myocyte cross-sectional areas, 6-μm thick OCT-embedded sections were prepared from hearts and subsequently stained with Alexa Fluor 488 conjugated-WGA (VECTOR) for cell border determination. The images were captured and then analyzed by Image J software.

**TUNEL staining**

6-μm thick OCT-embedded heart tissue sections were used for TUNEL staining with TUNEL Apoptosis Detection Kit (Alexa Fluor 640; 40308ES, Yeasen Biotechnology, Shanghai, China). Briefly, the frozen sections were warmed back to room temperature and fixed with 4% paraformaldehyde solution. Then, the slides were treated with proteinase K (20 μg/ml) for 15 min at room temperature. Next, the slide was preliminarily incubated with the TdT incubation buffer (50μ system) containing ddH2O, 5×Equilibration Buffer, Alexa Fluor 640-12-dUTP Labeling Mix, and recombinant TdT Enzyme at 37℃ in dark for 1h. The cardiomyocytes were viewed and photographed using a confocal fluorescence microscope.

**RNA sequencing and data analysis**

Whole-genome gene expression analysis was performed using the heart tissues from DOX-treated (n = 5), DOX with PrA-treated (n = 4) and saline-treated mice (n = 4). The total RNA was extracted using Trizol (Invitrogen) and stored at -80 °C. RNA isolation, quality control, library construction, and sequencing were performed by the Shanghai OE Biotech Co., Ltd (www.oebiotech.com/). A P value < 0.05 and a fold change ≥ 1.5 were set as thresholds for significant differential expression. The hierarchical cluster analysis of differentially expressed genes (DEGs) proved the expression patterns of genes in different groups and samples. All the differentially expressed genes were used for heat map analysis. KEGG enrichment analysis was performed on DEGs based on the hypergeometric distribution algorithm to screen for significant enrichment functional entries. GSEA was performed using cluster Profiler package. All codes are available from the corresponding author upon request.

**Quantitative real-time PCR**

Total RNA was isolated from heart tissues, H9c2 cells, or primary cardiomyocytes using TRIzol™ reagent (Invitrogen, Carlsbad, CA, USA) and reverse-transcribed using Transcriptor First Strand cDNA Synthesis Kit (Roche Diagnostics, Risch-Rotkreuz, Switzerland), following the manufacturer’s protocol. Quantitative real-time PCR was performed using a CFX96 Real-Time System (Bio-Rad) and Fast Start Universal SYBR® Green Master Mix (Roche Diagnostics) in accordance with the manufacturer’s instructions. The mRNA expression levels detected in each sample were normalized to β-actin levels. The forward and reverse primer sequences are listed as Supplementary Table S1.

**Western blot analysis**

Total protein was extracted from mice heart tissues, H9c2 cells, or primary cardiomyocytes. The protein content was then determined by using a BCA protein assay kit (P0012, Beyotime, Shanghai, China). The homogenate was cleared by centrifugation at 4°C for 30 min at 12,000 rpm, and the supernatant (containing the protein fraction) was collected. After denaturing by boiling for 5 min, cell and tissue lysates were separated by 12.5% SDS-PAGE and electro-transferred to a PVDF membranes. After blocking for 40 min in Protein Free Rapid Blocking Buffer (21I06, Epizyme). Membranes were incubated overnight at 4°C with primary antibodies. Next, the membranes were incubated with the secondary antibodies for 1 hour at room temperature. A Tanon Image software (version 5100; Tanon, Shanghai, China) was utilized to visualize the band signals. Densitometric quantification was performed using Image J analysis software. The signal intensities of the target bands were normalized to β-actin and then calculated as fold changes relative to the control groups. The antibodies are listed as Supplementary Table S2.

**Immunofluorescence staining**

**In vivo:** Heart issues were embedded within an OCT and cut to 6μm. The frozen sections were fixed with 4% paraformaldehyde solution after being warmed back to room temperature. Subsequently, the sections were permeabilized in 0.5% Triton X-100 (Sigma), blocked with 5% bovine serum albumin (BSA) for 30 minutes, and incubated with primary antibodies overnight at 4℃. Next, antibodies were detected by fluorescent-conjugated secondary antibodies for 1 hour. Nuclei were stained with 4′,6–Diamidine–2′– phenylindole dihydrochloride (DAPI, Beyotime, China).

**In vitro:** Cardiomyocytes were incubated as indicated. Following incubation, they

were washed with PBS and fixed with 4% paraformaldehyde (PFA) for 10 minutes. After blocking with 5% BSA for 30 min, they were incubated with primary antibodies at 4°C overnight. Next, antibodies were detected by fluorescent-conjugated secondary antibodies for 1 hour. After washing, the cells were stained with DAPI. A confocal fluorescence microscope was used to observe the location and distribution of immunoreactive substances in heart tissue or cells. The antibodies are listed in Supplementary Table S2.

**DHE staining**

The level of ROS in heart tissues was determined using a reactive oxygen species assay kit (BB-470516, Bestbio, Nanjing, China). Briefly, the cryosections were washed with PBS and incubated with DHE for 30 min at 37 °C in the dark. DHE fluorescence intensity was measured using a confocal fluorescence microscopy at excitation and emission wavelengths of 535 nm and 610 nm, respectively.

**Cell viability assay**

H9c2 cells and primary cardiomyocytes were seeded at 10^3^ ~10^4^ cells into a 96-well opaque plate. After the indicated treatment, cell viability assay was performed using the Cell Counting Kit reagent (CCK-8; CK04, F374, Dojindo Laboratories, Shanghai, China), according to the manufacturer’s instructions. The luminescent signal at the wavelength of 450 nm was measured. According to the recorded luminescence, the percentage of cell viability was then calculated.

**Apoptosis Assay**

The cell viability of H9c2 cells and primary cardiomyocytes was detected by FDA/PI double-stained cell viability detection kit (Kanglang Biotechnology, Shanghai, China). Cultured cells in 6-well plates were washed with PBS and stained with FDA at 37℃ in the dark for 20 min. Subsequently, cells were incubated with PI at 4℃ in the dark for 5 min. Staining images were taken with a fluorescence microscope.

**Flow cytometry-based lipid peroxidation assay**

Cells were seeded at 10^6^ cells per well in a 6-well plate and treated as indicated. After 24 hours of treatment, the cells were incubated in 10µM C11-BODIPY581/591 (RM02821, ABclonal Technology Co., Ltd) for 1h at 37°C in the dark. After removing excess C11-BODIPY and washing the cells twice with PBS, labeled cells were trypsinized and resuspended in PBS plus 5% FBS. Oxidation of the polyunsaturated butadienyl portion of C11-BODIPY resulted in a shift of the fluorescence emission peak from ∼590 nm to ∼510 nm proportional to lipid ROS generation and was analyzed using a flow cytometer.

**DCFH-DA staining**

The level of intracellular ROS was analyzed by a reactive oxygen species assay kit (S0033, Beyotime Biotechnology, Shanghai, China). Briefly, cells were inoculated into 6-well plates with 1 ml medium and treated as indicated. Then cells were incubated with DCFH-DA (10 μmol/L) in the dark at 37°C for 30 minutes. DCF fluorescence intensity was measured using a fluorescence microscopy at excitation and emission wavelengths of 488 nm and 525 nm, respectively.

**Measurements of iron in cytoplasm and mitochondria**

Intracellular ferrous ion (Fe^2+^) was measured using a FerroOrange fluorescence probe (F374, Dojindo Laboratories, Shanghai, China). Briefly, H9c2 cells were treated as indicated. After being washed with Hank’s Balanced Salt Solution (HBSS) twice, cells were incubated with 1 μM FerroOrange at 37 ℃ for 30 min. Next, the fluorescence of FerroOrange was detected at the excitation/emission wavelength of 561/570-620 nm using a confocal fluorescence microscopy.

Mitochondrial ferrous ion (Fe^2+^) was measured using a Mito-FerroGreen fluorescence probe (MFG; F489, Dojindo). After being treated as indicated, H9c2 cells were incubated in 5 μM MFG solution with 0.1 μM MitoBright Deep Red (MT12, Dojindo) for 30 minutes at 37°C in the dark. Next, mitochondrial iron was observed (an excitation wavelength of 488 nm and an emission wavelength of 500-550 nm) using a confocal fluorescence microscopy.

**Measurement of mitochondrial lipid peroxidation**

Lipid peroxidation (LPs) in the mitochondrial inner membrane was measured using MitoPeDPP fluorescence probe (M466, Dojindo). After being treated as indicated, H9c2 cells were incubated in the presence of 0.5μM MitoPeDPP solution with 0.1μM MitoBright Red (MT11, Dojindo) for 30 minutes at 37°C. Following HBSS washes twice, LPs in the mitochondrial inner membrane were measured (an excitation wavelength of 470/40 nm and an emission wavelength of 525/50 nm), using a confocal fluorescence microscopy.

**Measurement of mitochondrial Superoxide**

ROS in the mitochondrial was measured using a mtSOX Deep Red fluorescence probe (MT14, Dojindo). After being treated as indicated, H9c2 cells and primary cardiomyocytes were incubated in the presence of 10 μM mtSOX Deep Red solution for 30 minutes at 37°C. Following HBSS washes twice, ROS in the mitochondrial was measured (an excitation wavelength of 633 nm and an emission wavelength of 640-700 nm), using a confocal fluorescence microscopy.

**Analysis of mitochondrial membrane potential (ΔΨm)**

ΔΨm was measured using a Mitochondrial Membrane Potential Assay Kit (M8650, Solarbio, Beijing, China), following the manufacturer’s instructions. Briefly, after H9c2 cells and primary cardiomyocytes were treated as indicated, the cells were incubated with JC-1 working solution at 37 °C for 20 min. Subsequently, JC-1 buffer solution was used to wash cells at least two times. Red emission of the dye represented a potential-dependent aggregation in the mitochondria, reflecting ΔΨm. Green fluorescence represented the monomeric form of JC-1, appearing in the cytosol after mitochondrial membrane depolarization. Results were analyzed as the ratio of the green/red fluorescence intensity using Image J analysis software, which represented the degree of mitochondrial damage.

**Transmission electron microscopy**

Murine heart tissue samples were fixed in 3% phosphate-glutaraldehyde and stored at 4 ^◦^C in the dark overnight. The sections were dehydrated and stained with lead citrate and uranyl acetate for measurement. Finally, the samples were viewed, using a transmission electron microscope (HITACHI, H-7650, JAPAN).

**Functional Enrichment Analysis**

Kyoto Encyclopedia of Genes and Genomes (KEGG) pathway enrichment analysis was performed on PrA-binding proteins.

**Protein–protein Interaction (PPI) Network**

The Search Tool for Recurring Instances of Neighboring Genes (STRING) system, a database of protein-protein interactions, was used to build the biological interaction networks for PrA binding proteins which enrichment in Ferroptosis-related pathways, including Ferroptosis, Glutathione metabolism, and peroxisome.

**Pull-down assay**

For the pull-down assays, we used Pierce^TM^ Pull-Down Biotinylated Protein Interaction kit (Thermo Fisher). 100 µL of 50ug/100ul biotinylated-PrA was added to 50 μL streptavidin-agarose beads and incubated at 4°C for 30 minutes. Biotin alone was used as a control. Lysates more than 100ul prepared from H9c2 cells were then added to the streptavidin-agarose beads with bio-PrA. The mixture was incubated at 4°C for 24 hours with gentle rocking. Samples were then spun and washed 3 times with Wash Buffer. Before elution, 10µL of the Neutralization Buffer was added to each elution Collection Tube to neutralize the pH of the contents upon elution. Subsequently, 250µL of Elution Buffer was added to each spin column. The top screw caps were secured onto the columns, and the solution was mixed by gently inverting the columns 5-7 times. The spin column was incubated for 3-5 minutes at room temperature and centrifuged at 1250 ×g for 30-60 seconds. Eluent was collected and boiled with 5x loading buffer. Western blotting was developed to check the pull-down bands. Total lysates were used as an input control.

**Molecular docking and dynamics simulation**

The crystal structure of ACSL4 and FTH1 were derived from Protein Data Bank and the UniPort database. The 3D structure of PrA was downloaded from the PubChem database and optimized using the MMFF94 force field of OpenBabel software to obtain the optimal molecular structure with the lowest energy state. Before testing, the structures of ACSL4, FTH1, and PrA were ensured to be in the optimized active arrangement. Finally, molecular docking was performed using Auto Dock Vina1.2.0.

Molecular dynamics simulation was performed using Gromacs2022 software.

In order to restore the real experimental environment, Charmm 36 and Gaff 2 were selected as the protein force field and the ligand force field, respectively, while TIP 3 P water model was selected to add solvent to the protein ligand system. Then, a water box with periodic boundary of 1.2nm was established, and sodium ions and chloride ions were added to equilibrize charges in the system. After assignment of force field to the protein and a series of energy minimizations, the simulation was performed.

**ACSL4 knockdown and overexpression**

Gene silencing in cells was achieved by transfecting cells siRNA (Hanheng Reagent, Shanghai, China). Custom siRNAs were synthesized for rat ACSL4 (5’-GCAGAGTGAATAACTTTGGAA-3’, 5’-GCAGAAGATTATTGTGTTGAT-3’ and 5’-GCCATGAAATTGGAGCGATTT-3’). Control cells were transfected with negative control siRNA. ACSL4 overexpression in cells was implemented by transfecting cells with ACSL4 plasmids (pcDNA3.1-CMV-mcs-3flag-EF1a-puro; Hanheng Reagent, Shanghai, China).

**Cellular thermal shift assay**

293 T cells were transfected with plasmids of pcDNA3.1-3×Flag-h-ACSL4 (P445A, I567A) -3×Flag-EGFP and pcDNA3.1-3×Flag-h-FTH1(R23A, Q84A)-3×Flag-EGFP using lipo2000 for 48 h, with the empty vector selected as a control. After incubating with PrA or DMSO for 6 h, the cells were collected in PBS containing 1% protease inhibitor and divided into ten groups. The samples were heated at a series of temperatures ranging from 37 to 64 °C, with a gradient of 3 °C for 3 min each. After being frozen by using liquid nitrogen and thawed on ice twice, the supernatant cell lysates were collected for the western blotting.

**Measurement of Arachidonic acid (AA)**

AA was tested by ELISA kit (JLC78180, Jingkang Biotechnology, Shanghai, China) as described in the protocol.

**Flow cytometry analysis of apoptosis**

Flow cytometry analysis of apoptosis was detected by Annexin V-FITC/PI Apoptosis Detection kit (40302ES, Yeasen Biotechnology, Shanghai, China). Briefly, the cells were washed twice with cold PBS and resuspended in the binding buffer. Each sample was calculated using 10^6^ cells (100 μl) and incubated with 5 μl of Annexin V-FITC and 10 μl of propidium iodide (PI) for 15 minutes in the dark. Subsequently, 400 μl of binding buffer was added to each sample. The percentage of positive cells was analyzed using a FACSCanto II flow cytometer (BD Biosciences, CA, USA).

**Co-immunoprecipitation (Co-IP)**

# Co-IP was performed with Pierce Crosslink Magnetic IP/Co-IP Kit (abs955, Absin, Shanghai, China). Briefly, the cell lysate was incubated with anti-FTH1 (4393, Cell Signaling Technology, Shanghai, China) for 24 h at 4 °C. Subsequently, protein A/G Agarose was added and incubated for 3h at 4 °C followed by centrifuged at 12000rpm for 5 min. Next, the precipitate was recovered for washing 3 times, resuspended by 50 μl SDS lysis buffer, and boiled 5 minutes for immunoblotting with the indicated antibody. The total protein from cell lysates was used as the positive control.

**Measurements of iron in lysosome**

The H9c2 cells were seeded in 24-well plates and grew overnight. After being treated as indicated in the main figure, cells were co-stained with FerroOrange (1 μM) and LysoTracker Green (50 nM) in HBSS for 30 min at 37 °C in the dark and washed with PBS. Finally, the digital images were recorded using a confocal fluorescence microscopy and analyzed with Image J software.

**2,3,5-triphenyltetrazolium chloride (TTC)-Evans blue double staining**

To measure the infarct size, the mice were re-anesthetized 24 h after I/R surgery and injected with Evans blue dye (0.5%; G1810, Solarbio) via external iliac vein after the occlusion of the left anterior descending artery. Then, the heart was excised and rinsed in PBS. The tissue was frozen at -20 °C for 20 min and evenly sectioned from the ligation to the apex (1 mm thickness). Next, the sections were incubated with 2% TTC (G3005, Solarbio) at 37° C for 15 min to visualize the unstained infarcted region, and they were digitally photographed. The remote area was stained blue, the area at risk was stained red (AAR), and the infarct area was visualized using unstained white (IF). The infarct size, expressed as a percentage of AAR, was calculated using Image J software.

**Supplementary Figure S1. PrA ameliorates DOX-induced cardiac injury in female mice.** A) Kaplan-Meier survival curves of C57BL/6 female mice in each group (n = 20 per group). B-F) Representative echocardiographic images and quantitative analysis of LVEF, FS, LVIDd, and LVIDs (n = 6 per group). G-H) Serum CK-MB (5-fold dilution) and LDH levels were measured in each group (n = 6 per group). I) Relative mRNA levels of Ptgs2 in murine hearts (n = 6 per group). J-M) Cardiac protein expression of GPX4, ACSL4, and FTH1 was measured by immunoblotting (n = 6 per group). N) Representative images of H&E staining (n = 6 per group, scale bar = 100 μm). O-P) Representative images (O) and quantitative analysis (P) of Masson’s trichrome staining (n = 6 per group; scale bar = 100 μm). Some of the data was normalized (H-I, K-M). Summary data are presented as the mean ± SEM. Statistical significance was calculated using the log-rank (Mantel–Cox) test (A) and one-way ANOVA with Tukey's multiple comparisons test (C-I, K-M, P). *P < 0.05, **P < 0.01, ***P< 0.001, ****P< 0.0001.

Abbreviations: CK-MB, creatine kinase-MB; DOX, doxorubicin; FS, left ventricular fractional shortening; LDH, lactate dehydrogenase; LVEF, left ventricular ejection fraction; LVIDd, left ventricular internal dimension in diastole; LVIDs, left ventricular internal dimension in systole; PrA, Protosappanin A, SEM, standard error of mean.

**Supplementary Figure S2. PrA inhibits DOX-induced cardiac ferroptosis.** A) Volcano plot representing regulated genes with fold change. B) Venn diagram representing the screened genes in the intersection part with A. C) KEGG pathway enrichment analysis in DOX-treated murine heart with or without PrA. D-F) The protein quantification of GPX4 (D), ACSL4 (E), and FTH1 (F) in control mice and DOX-treated (20 mg/kg, i.p.) mice with or without PrA (5 or 20mg/kg, i.g.; n = 6 per group). G-H) The quantification of GPX4 (G), ACSL4 (H), and FTH1 (I) fluorescence intensity in control mice and DOX-treated (20 mg/kg, i.p.) mice with or without PrA (5 or 20mg/kg, i.g.; n = 6 per group). Some of the data was normalized (D-H). Summary data are presented as the mean ± SEM. Statistical significance was calculated using one-way ANOVA with Tukey's multiple comparisons test. **P* < 0.05, ***P* < 0.01, ****P* < 0.001, *****P* < 0.0001.

Abbreviations: DOX, doxorubicin; i.p., intraperitoneal; i.g.,intragastric; PrA, Protosappanin A, SEM, standard error of mean.

**Supplementary Figure S3. PrA mitigates DOX-induced iron accumulation and lipid peroxidation and improves mitochondrial function.** A) The effect in different concentrations of PrA for 24 h, cell viability was detected by CCK8 assay (n = 8 per group). B-D) Western blot analyzed GPX4 (B), ACSL4 (C), and FTH1 (D) in H9c2 cells after treatment of different PrA concentrations (n = 6 per group). E) Relative quantitative fluorescence intensity analysis of reactive oxygen species (ROS) (n = 6 per group). F) Relative quantitative fluorescence intensity analysis of cytoplasmic ferrous ions (n = 6 per group). G, J-K) Representative fluorescence images (G) and the quantitative analysis(J-K) of JC-1 staining with mtSOX Deep Red staining (scale bars: 20 μm; n = 6 per group). H, L) Representative fluorescence images (H) and the quantitative analysis (L) of mitochondrial iron using Mito-FerroGreen (green, MFG; purple, MitoBright LT Deep Red; scale bars: 20 μm; n = 6 per group). I, M) Representative fluorescence images (I) of mitochondrial LPs using MitoPeDPP and quantitative analysis (M) of fluorescence intensity to determine levels of LPs in H9c2 cells (green, MitoPeDPP; red, MitoBright LT Deep Red; scale bars: 20 μm; n = 6 per group). All data were normalized. Summary data are presented as the mean ± SEM. Statistical significance was determined using multiple unpaired 2-tailed Student t-tests (A) and one-way ANOVA with Tukey's multiple comparisons tests (B-F, J-L). **P* < 0.05, ***P* < 0.01, ****P*< 0.001, *****P*< 0.0001.

Abbreviations: DOX, doxorubicin; PrA, Protosappanin A, SEM, standard error of mean.

**Supplementary Figure S4. PrA protects primary cardiomyocytes against DOX-triggered cellular damage.** A) The effect in different concentrations of DOX-induced cell death for 24 h; cell viability was detected using CCK8 assay (n = 6 per group). B) The effect in different concentrations of PrA for 24 h; cell viability was detected using CCK8 assay (n = 8 per group). C-D) Representative images of PI staining (C) and the percentage of PI-positive cells (D) (black and white: phase contract; red: PI staining, scale bar = 100 μm; n = 6 per group). E) Relative mRNA levels of Ptgs2 (n = 6 per group). F-I) Protein expression of GPX4, ACSL4, and FTH1 was measured using immunoblotting (n = 6 per group). J-L) Representative fluorescence images (J) and the quantitative analysis (K-L) of JC-1 staining with mtSOX Deep Red staining (scale bar = 10 μm; n = 6 per group). Some of the data was normalized (A-B, E, G-I, K-L). Summary data are presented as the mean ± SEM. Statistical significance was determined using multiple unpaired 2-tailed Student t-tests (A-B) and one-way ANOVA with Tukey's multiple comparisons tests (D-I, K-L). **P* < 0.05, ***P* < 0.01, ****P*< 0.001, *****P*< 0.0001.

Abbreviations: DOX, doxorubicin; PrA, Protosappanin A, SEM, standard error of mean.


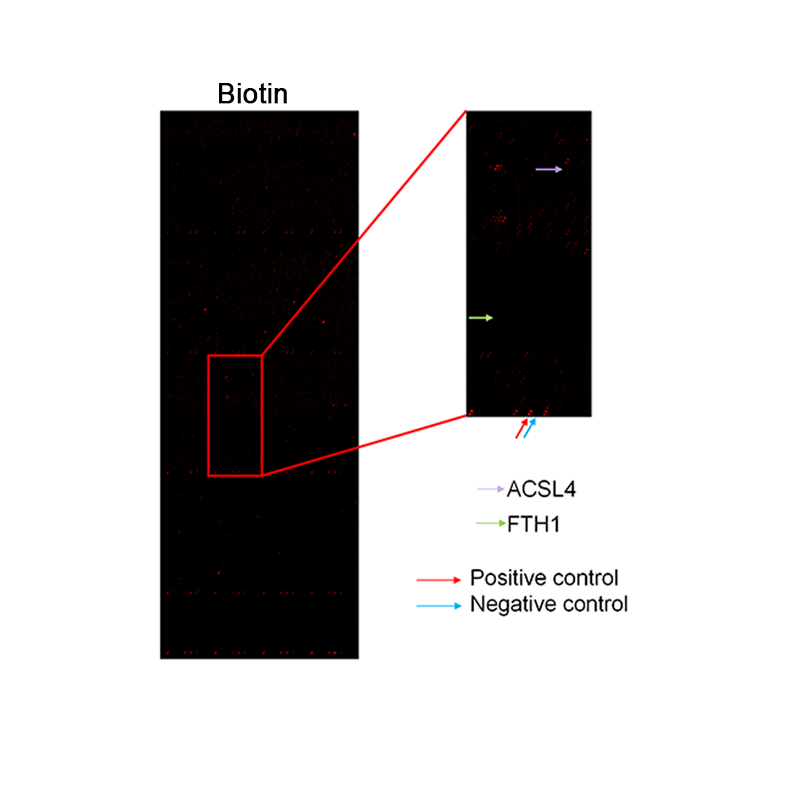


**Supplementary Figure S5. Representative image of protein array without PrA as a control experiment.** The images show positive (red arrow) and negative control (blue arrow) spots, as well as spots for ACSL4 (purple arrow) and FTH1 (green arrow)**.**

**Supplementary Figure S6. PrA inhibits DOX-induced ACSL4 threonine 328 phosphorylation.** A-B) Protein quantification analysis of p-ACSL4 (A) and ACSL4 (B) in mice heart tissues (n = 6 per group). C) Protein quantification analysis of ACSL4 in H9c2 cells exposed to 1 µM DOX for different times (n = 6 per group). D-E) Protein quantification analysis of p-ACSL4 (D) and ACSL4 (E) in H9c2 cells after treatment of different PrA concentrations (n = 6 per group). F-G) Protein quantification analysis of p-ACSL4 (F) and ACSL4 (G) in H9c2 cells following ACSL4 knockdown (n = 6 per group). H-I) Protein quantification analysis of ACSL4 (H) and p-ACSL4 (I) in H9c2 cells following ACSL4 over expression (n = 6 per group). All data were normalized. Summary data are presented as the mean ± SEM. Statistical significance was determined using one-way ANOVA with Tukey's multiple comparisons test. **P* < 0.05, ***P* < 0.01, ****P* < 0.001, *****P* < 0.0001.

Abbreviations: DOX, doxorubicin; PrA, Protosappanin A, SEM, standard error of mean.

**Supplementary Figure S7. The inhibitory effects of PrA on ischemia/reperfusion (I/R)-induced ferroptosis.** A) Quantitative analysis of cardiac infarct size per area at risk (n = 6 per group). B-D) Protein quantification analysis of GPX4 (B), ACSL4 (C) and FTH1(D) (n = 6 per group). E) Quantification of size for Prussian blue staining with DAB enhancement (n = 6 per group). F) Quantification of fluorescent immunohistochemistry staining for DHE (n = 6 per group). Some of the data was normalized (B-F). Summary data are presented as the mean ± SEM. Statistical significance was determined using one-way ANOVA with Tukey's multiple comparisons test. **P* < 0.05, ***P* < 0.01, ****P* < 0.001, *****P* < 0.0001.

Abbreviations DOX, doxorubicin; PrA, Protosappanin A, SEM, standard error of mean.

**Supplementary Table S1. Primer pairs for Quantitative real-time PCR**

| **Gene** | **Primer Sequence (5’->3’)** |
| --- | --- |
| Rat ptgs2 Forward | TGCATTCTTTGCCCAGCACT |
| Rat ptgs2 Reverse | ACCTCTCCACCAATGACCTGAT |
| Rat FTH1 Forward | GGAGCATGCCGAGAAACTGA |
| Rat FTH1 Reverse | AGTCATCACGGTCTGGTTTCTTT |
| Rat β-actin Forward | GAGGTATCCTGACCCTGAAGTA |
| Rat β-actin Reverse | CACACGCAGCTCATTGTAGA |

**Supplementary Table S2. Antibodies used for immunoblotting and immunofluorescence**

| **Antibodies** | **Source** | **Indentifier** |
| --- | --- | --- |
| FTH1（D1D4） Rabbit mAb | Cell signaling | Cat#4393s |
| Anti-FACL4 antibody | Abcam | Cat#ab155282 |
| Anti-Glutathione Peroxidase 4 antibody | Abcam | Cat#ab125066 |
| Anti-NCOA4 antibody | Abcam | Cat#ab86707 |
| Anti-Cardiac Troponin T antibody [1C11] | Abcam | Cat#ab8295 |
| CD107b / LAMP2 Monoclonal antibody | Proteintech | Cat#66301-1-Ig |
| Anti-LC3B Rabbit mAb | PTM Bio | Cat#PTM-6384 |
| Anti-beta Actin Rabbit mAb | PTM Bio | Cat#PTM-5028 |
| Mouse Anti-β actin mAb | ZSGB-BIO | Cat# TA-09 |
| Goat Anti‐ Rabbit IgG H&L | ZSGB-BIO | Cat#ZB-5301 |
| Goat Anti‐ Mouse IgG H&L | ZSGB-BIO | Cat#ZB-2305 |
| Goat Anti‐ Mouse IgG H&L | ZSGB-BIO | Cat#ZB-2305 |
| Goat Anti‐ Rabbit IgG H&L | Abcam | Cat#ab150081 |
| Donkey Anti‐ Mouse IgG H&L | Abcam | Cat#ab150116 |
